# Depletion of CX3CR1^+^ macrophages results in disrupted functionality and immune surveillance within epididymis and testis

**DOI:** 10.1016/j.mucimm.2026.01.011

**Published:** 2026-01-30

**Authors:** D. Ai, L. Kreyling, M.A. Battistone, M.L. Elizagaray, A. Chen, S. Bhushan, M. Fijak, M. Speckmann, G. Michel, T. Procida-Kowalski, M. Bartkuhn, M. Sprang, J.U. Mayer, A. Meinhardt, C. Pleuger

**Affiliations:** a Department of Anatomy and Cell Biology, Unit of Reproductive Biology, Justus-Liebig University Giessen, Germany; b Hessian Centre of Reproductive Medicine, Justus-Liebig University Giessen, Germany; c Program of Membrane Biology, Nephrology Division, Department of Medicine, Massachusetts General Hospital and Harvard Medical School, Boston, MA, USA; d Flow Cytometry Core Facility, Justus-Liebig University Giessen, Germany; e Biomedical Informatics and System Medicine, Science Unit for Basic and Clinical Medicine, Justus-Liebig University Giessen, Germany; f Department of Dermatology, University Medical Center of the Johannes Gutenberg-University Mainz, Mainz, Germany; g Research Center for Immunotherapy (FZI), University Medical Center of the Johannes Gutenberg-University Mainz, Mainz, Germany; h Institute of Quantitative & Computational Biosciences, Johannes Gutenberg University Mainz, Mainz, Germany

**Keywords:** Male reproductive tract, Epididymis, Testis, CX3CR1^+^ macrophages, Mononuclear phagocytes, Mucosal immunology, Immune regulation, Sperm function, Spermatogenesis, Epithelial physiology, Epididymitis, UPEC

## Abstract

A finely tuned immune regulation within the epididymis and testis is essential for male reproductive health. This balance is especially critical in the epididymis, where sperm mature and ascending infections frequently disrupt homeostasis, resulting in regionally different immune responses and potential long-term fertility impairments. We previously demonstrated that the epididymis harbors a region-specific immunological scaffold, with CX3CR1^+^ macrophages as the most prominent epithelium-associated immune cell population. Here, we established a transgenic mouse model to selectively deplete these intraepithelial CX3CR1^+^ macrophages within the epididymis, resulting in focal epithelial damage and impaired sperm maturation processes essential for proper sperm functionality. Additionally, a mild reduction of the testicular macrophage pool resulted in transient disruptions in spermatogenesis and steroidogenesis. Although the macrophage niche was repopulated after depletion, the newly recruited cells displayed altered phenotypes consistent with persistent sperm alterations. Following infection with uropathogenic Escherichia coli (UPEC), macrophage-depleted mice exhibited exacerbated immune responses – particularly in normally protected proximal epididymal regions – with earlier onset and more severe tissue damage. Transcriptomic analysis revealed a failure to restrain inflammatory responses, especially in genes involved in immune regulation and antibacterial defense, accompanied by elevated immune cell infiltration in infected macrophage-depleted mice. Overall, our findings confirm a crucial role for CX3CR1^+^ macrophages in preserving epithelial integrity and modulating immune responses, supporting a stable tissue environment necessary for efficient organ function of both epididymis and testis.

## Introduction

Male fertility relies on the coordinated function of the testis and epididymis—adjacent organs with distinct roles in sperm production and maturation. Although both organs are exposed to similar antigens and immunological challenges, they exhibit markedly different immune architectures. Spermatogenesis occurs within the immune-privileged environment of the testis, whereas spermatozoa, once released, encounter the immune system in the epididymis, where essential post-testicular maturation takes place. To prevent autoimmune reactions against autoantigenic spermatozoa the immune system must establish a tolerogenic milieu within the epididymis while retaining the capacity to mount effective immune responses against ascending urogenital pathogens.

Disruption of this delicate immunological balance – most often triggered by acute bacterial infections^1^ – often results in long-term fertility impairments. Indeed, up to 40% of patients with epididymitis experience persistent sub- or infertility,^2^ with a clear correlation between epididymitis and the occurrence of anti-sperm antibodies.^3^ Experimental mouse models have shown that epididymal tissue damage is primarily driven by an exacerbated proinflammatory immune response rather than bacterial presence *per se*.^4–6^ Notably, these immune responses manifest most severely in the cauda epididymidis, leading to duct stenosis/obstruction and fibrotic remodeling,^7,8^ whereas in proximal regions inflammation is thoroughly controlled and rapidly resolved, preserving tissue integrity.^6^

Recent studies have highlighted the epididymis as a mucosal tissue with a regionally specialized immune architecture. Resident immune cells, including both myeloid and lymphoid populations, are strategically positioned along the duct,^6,9,10^ occupying specific niches that reflect regional demands for tissue homeostasis, sperm tolerance and immune defense. Among these, CX3CR1^+^ mononuclear phagocytes, with a macrophage signature, represent the predominant immune cell population and are primarily located within and around the epididymal epithelium.^6,10–12^ Particularly within the initial segment, the entry point for sperm, intraepithelial CX3CR1^+^ macrophages extend long protrusions between neighboring epithelial cells toward the lumen, suggesting a crucial sentinel role in monitoring luminal contents.^10,11^

Despite their strategic positioning and unique morphology, the functional role of CX3CR1^+^ macrophages in maintaining epididymal immune balance remains incompletely understood. Transcriptomic profiling reveals that epididymal CX3CR1^hi^ macrophages typically display a homeostatic transcriptional signature, in contrast to the more proinflammatory profile of interstitial macrophage subsets.^6^ Across different epididymal regions, CX3CR1^hi^ macrophages become less prevalent and adopt a more pro-inflammatory signature towards the distal end,^10^ consistent with the regiońs heightened vulnerability to pathogen invasion and tissue damage. These observations point to a dual role for CX3CR1^+^ macrophages within the epididymis – supporting epithelial integrity and sperm maturation within proximal regions, while serving as immune sentinels in distal regions. However, whether these cells are causally required for maintaining regional immune homeostasis and the consequences from their absence, have not been systematically examined.

Within the testis, macrophages substantially contribute to the establishment and maintenance of the immune-privileged environment that allows germ cells to develop without eliciting immune responses.^13^ By actively suppressing and resolving inflammation (e.g. in response to ascending bacterial infections), they help preventing immune-mediated damage to developing gametes. Although testicular macrophages also (partially) express CX3CR1, they are functionally and transcriptionally distinct^5^ and are restricted to the interstitial space, in contrast to the more epithelium-associated localization observed in the epididymis.

In this study, we selectively depleted CX3CR1^+^ macrophages *in vivo* in a transgenic mouse model to investigate their role in regulating immune homeostasis in the epididymis and testis under physiological conditions. Given that macrophages in both the epididymis and testis express CX3CR1 and have been implicated in supporting sperm development and maturation,^14,15^ we evaluated the impact of CX3CR1^+^ macrophage depletion on the local immune system and fertility-related physiological parameters in both organs. To further examine the functional consequences of macrophage loss during infection, we employed an established mouse model of acute bacterial epididymitis that closely mimics the clinical features observed in patients^16^ and analyzed region-specific immunity following infection with uropathogenic Escherichia coli (UPEC).

## Materials and methods

### Experimental mice

Mice used in this study were bred and housed under specific pathogen-free conditions in individually ventilated cages with frequent air changes (15/h). Animals were kept under a 12 h light/dark cycle with ambient temperature (22 ± 2°C) and a relative humidity of 55 ± 10%. The following strains were obtained from the Jackson Laboratory: *Cx3cr1*^CreER^ (B6.129P2(C)-*Cx3cr1*^*tm2.1(cre/ERT2)Jung*^/J, JAX #020940,^17^ *Rosa26*^iDTR^ (C57BL/6-*Gt(ROSA)26Sor*^*tm1(HBEGF)Awai*^/J, JAX #007900,^18^ and *Rosa26*^tdTomato^ (B6.Cg-*Gt(ROSA)26Sor*^*tm9(CAG-tdTomato)Hze*^/J, JAX #007909.^19^ Mice were maintained according to the supplier’s instructions at the animal facility of the Justus-Liebig University Giessen, Germany. All animal experiments were approved by the local Animal Ethic Committee (Regierungspräsidium Giessen GI20/25 No. G58/2020 and No. G62/2021) and performed in strict accordance with the Guidelines of the Care and Use of Laboratory Animals of the German law for animal welfare and the European legislation for the protection of animals for scientific purposes (2010/63/EU). For euthanasia before organ collection, mice were deeply anaesthetized by inhalation of 4–5% isoflurane, followed by cervical dislocation or terminal heart puncture.

### Depletion of CX3CR1^+^ cells

**Fig. 1.**
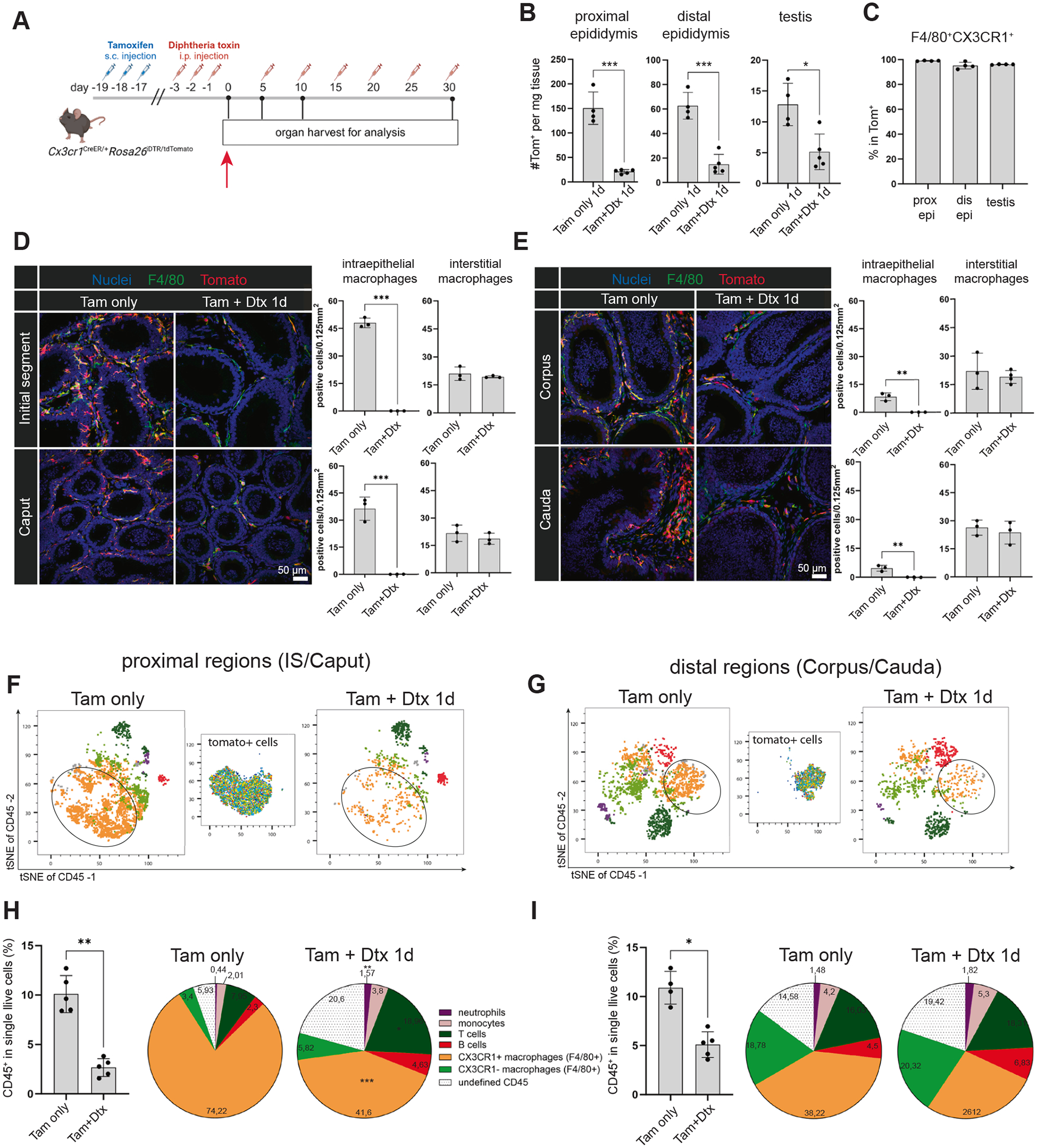
Targeted depletion of intraepithelial CX3CR1^+^ macrophages in the murine epididymis. (A) Schematic overview of the depletion strategy using sequential tamoxifen (Tam) and diphtheria toxin (Dtx) treatment in *Cx3cr1*^CreER/+^*Rosa26*^iDTR/tdTomato^ mice. (B) Flow cytometry-based quantification of tdTomato^+^ (Tom^+^) cells per mg tissue in proximal (IS/ caput) and distal (corpus/ cauda) epididymis, and testis following Dtx-mediated depletion, assessed by flow cytometry one day after depletion. (C) Proportion of F4/80^+^CX3CR1^+^ cells among Tom^+^ cells (each data point represents pooled proximal and distal regions from left and right epididymis, n = 4–5). (D, E) Confocal microscopy images of Tom^+^ cells (red) in intraepithelial and interstitial compartment of (D) proximal and (E) distal epididymal regions. Semi-quantitative analysis of Tom^+^ macrophages (F4/80^+^, green) was performed by counting ≥ 5 representative areas per mouse (n = 3). Nuclei were stained with DAPI (blue). (F, G) t-SNE plots (opt-SNE with iterations: 500, perplexity: 20) of CD45^+^ populations from representative Tam only and Tam + Dtx-treated mice, one day post depletion, in the (F) proximal and (G) distal epididymis. CD45^+^ cells were downsampled, gated as shown in Fig. S1F and overlaid onto the t-SNE plots. Tomato^+^ cells were gated separately and plotted on the t-SNE coordinates. Circles indicate differences in CX3CR1^+^ macrophages between Tam only and Tam + Dtx-treated mice. (H, I) Relative abundance of CD45^+^ cells and distribution of immune cell subsets (pie charts) within the CD45^+^ compartment in (H) proximal and (I) distal (G) regions of Tam only and Tam + Dtx-treated mice, one day post depletion. Immune cell populations are color-coded as in (F, G), based on the gating strategy in Fig. S1F. Mean values of indicated cell types are indicated in numbers [% in CD45^+^]. Statistical analysis was performed using the Kruskal–Wallis test with Dunn’s post hoc correction (*p < 0.05, **p < 0.005, ***p < 0.001). Data are presented as mean ± SD from two independent experiments (n = 4–5 mice). Statistical analysis was performed using Mann-Whitney *U* test, *p < 0.05, **p < 0.005, ***p < 0.001).

For experimental depletion of CX3CR1^+^ cells, *Cx3cr1*^CreER^ mice were crossed with *Rosa26*^iDTR^ mice. Compound homozygous males (*Cx3cr1*^CreER/CreER^*Rosa26*^iDTR/iDTR^) were further crossed with *Rosa26*^td-Tomato/tdTomato^ females to enable endogenous labeling of DTR-expressing cells upon tamoxifen treatment. To induce recombination, adult male *Cx3cr1*^CreER/+^*Rosa26*^iDTR/tdTomato^ mice (10–12 weeks old) received 125 μg/g body weight Tamoxifen (‘Tam’ [Sigma T5648] – dissolved in corn oil [Sigma C8267] by sonication for 15 min at 37°C. Tamoxifen was administered subcutaneously once daily for three consecutive days. To avoid a co-depletion of short-lived CX3CR1-expressing cells (i.e. neutrophils, monocytes), Diphtheria Toxin (‘Dtx’, [Sigma D0564] – dissolved in PBS) administration started no earlier than 14 days after the final Tam injection. Dtx was delivered by intraperitoneal injection acutely (500 ng daily on three consecutive days) followed by continuous application of 250 ng every fifth day. Mice were sacrificed at various time points after Dtx-mediated depletion for organ collection and downstream analyses. Day 1 after depletion was defined as the first day following the initial three consecutive Dtx injections (Fig. 1A).

### Induction of acute bacterial epididymitis

Uropathogenic *Escherichia coli* (UPEC) strain CFT073^20^ was cultured as described previously.^21^ To trigger acute epididymitis, mice were anaesthetized through ketamine/xylazine narcosis followed by a small incision at the scrotal area to expose the testis-epididymis-complex. After bilateral ligation of the vasa deferentia, 5 μl UPEC suspension (1–2 × 10^5^ colony forming units [CFU]) were injected into the vasa deferentia adjacent to the cauda epididymidis using a Hamilton syringe. Control ‘sham’ mice underwent the same experimental procedure with an intravasal injection of 5 μl 0.9% NaCl instead of bacteria. Mice were sacrificed by isoflurane inhalation and subsequent cervical dislocation. Each experimental group comprised 5–10 mice per time point, and the entire procedure, including downstream analyses, was repeated in at least two independent experiments.

### Determination of CFU

Testes and epididymides were collected 5d after UPEC infection and homogenized in 250 μl ice-cold sterile PBS. Serial dilutions were prepared and spread onto Luria broth (LB) agar plates (10 mg/ml tryptone, 5 mg/ml yeast extract, 10 mg/ml NaCl, 15 mg/ml agar agar, pH7.0) were incubated at 37°C for 24 h, after which CFU were determined and normalized to tissue weight. Pure UPEC cultures and sterile PBS (processed in parallel with tissue homogenates) were used as positive and negative controls, respectively.

### Histological evaluation

Dissected organs were fixed Bouińs solution for 6 h at room temperature (RT) before standard paraffin embedding. To improve fixative penetration, the testicular capsule was punctured with a 30G needle. Embedded specimens were cut into 5 μm sections, mounted onto Superfrost Plus slides (Thermo Scientific), incubated overnight at 37°C and stored at RT until further processing. Prior to all histological staining, sections were deparaffinized in xylene and rehydrated through a graded ethanol series. Testicular sections were stained with Periodic Acid Schif́fsreagent and hematoxylin. Epididymal section were stained using the Masson-Goldner trichrome method as described previously.^6^ Images were acquired using a Leica Thunder Imager and Leica DM750 microscope. Morphometric analyses were performed using ImageJ V1.53a.

### Scoring of histopathological alterations after UPEC infection

To categorize and compare histopathological alterations after UPEC infection, a previously described scoring systems for epididymitis and orchitis^6,22,23^ were adopted with minor modifications (Table 1).

### Assessment of testicular daily sperm production (DSP)

Decapsulated testes were homogenized in 1 ml DSP buffer (0.9% NaCl, 0.01% Azide, 0.05% Triton-X-100) using a bead-based homogenizer (Retsch). Homogenates were vortexed thoroughly and intact sperm heads counted using a hemocytometer (Buerker chamber 0.0025 mm^2^, depth 0.100 mm). Each homogenate was counted in duplicate, with counts obtained from five of the 16 squares per replicate. DSP was calculated as follows:

1. Average counted sperm × 50,000 (vol. of counted squares) × (vol. of homogenate [ml] + sample weight [g]) = n_sperm_/homogenate
2. n_sperm_/ homogenate/ sample weight [g] = n_sperm_/g testis
3. n_sperm_/ g testis × testis weight [g] = n_sperm_/ total testis
4. n_sperm_/ total testis /4.84 (duration of developing spermatids in step 14–16) = DSP/ testis

### Hormone level measurement

Serum was collected by terminal heart puncture and processed according to standard procedures. Testosterone concentrations were assessed using a commercially available mouse testosterone (Crystal Chem) and LH (ThermoFisher Scientific) ELISA kits with respective standardized positive controls, Appendix I, Table 2) following the manufactureŕs recommendations.

### Assessment of anti-sperm antibodies (ASA)

Proteins from untreated wildtype mice were obtained from sperm isolated from the cauda epididymidis. Sperm were incubated for 20 min on ice in RIPA buffer (supplemented with phosphatase and protease inhibitors), followed by ultrasonication and 30 min incubation on ice. Samples were centrifuged at 10,000 × g for 20 min, and the supernatant containing soluble proteins was collected to coat high-binding ELISA plates. Antigen solution (200 μl, 6 μg/ml) was incubated overnight at 4°C before washing with PBS-T. Plates were blocked with 3% BSA for 1 h at 37°C and then incubated for 2 h at 37°C with serum (1:50 in PBS containing 1% BSA) from untreated wildtype, Tam only and Tam + Dtx-treated mice. Uncoated wells treated with serum as well as coated wells treated with PBS served as controls for background reactions.

Bound IgG was detected by incubation with HRP-conjugated goat anti-mouse IgG antibody (0.2 μg/ml) for 2 h at RT. Following stringent washing with PBS-T, bound antibodies were visualized by adding TMB substrate for 20 min until the desired color developed. Reactions were stopped by adding 100 μl 2 M sulfuric acid, and optical density was measured at 450 nm in a microplate reader.

### Functional sperm analysis

Sperm were collected from the cauda epididymidis by back-flushing.^24,25^ and transferred in pre-warmed MT6 medium, allowing sperm to eqilibrate for at least 10 min at 37°C. A small fraction of the sperm suspension was collected as the T0 baseline control. Remaining sperm were incubated for 90 min in MT6 medium to induce capacitation. To trigger the acrosome reaction, 15 μM progesterone was added to the capacitation medium for the final 15 min of incubation. Sperm were then collected, washed in PBS and 50 μl aliquots of sperm drops were placed on SuperFrost Plus slides and air-dried overnight. Specimens were post-fixed with 10% neutral-buffered formalin for 15 min, followed by staining of phosphorylated tyrosine using an anti-4G10 antibody (1:1000, Appendix I Table 2). Sperm tails were visualized using an anti-acetylated tubulin antibody (1:100, Appendix I Table 2). Following overnight incubation with primary antibodies (4°C), corresponding secondary antibodies were applied for 1 h at RT. The acrosome (AR) was visualized with FITC-conjugated peanut agglutinin (PNA, 1 μg/ml) for 30 min at RT. Nuclei were stained using DAPI (5 μg/ml). Specimens were mounted using Invitrogen ProLong Gold Antifade Mountant and imaged using a Leica Stellaris 5 confocal microscope. For each biological replicate, at least 200 sperm were evaluated and categorized into (i) fully, partially and non-capacitated sperm according to the 4G10 staining pattern, and (ii) AR-reacted and AR-nonreacted according to the PNA-staining pattern (Suppl. Fig. S2D).

### Immunofluorescence

Tissues were fixed in 4% PFA for 6 h at 4°C followed by washing in phosphate buffer and overnight incubation in 30% sucrose before embedding in OCT. Sections (20 μm) were air-dried for 20 min at RT before staining. After washing in TBS + 0.05% Tween, pH 7.6 (TBS-T), sections were permeabilized using 0.2% Triton-X-100 and blocked using 3% BSA. Antigen retrieval was performed using either PBS containing 1% SDS (V-ATPase B1 subunit) or 1% SDS + 0.1% Triton-X-100 (aquaporin 9) for 4 min. Primary antibodies (Appendix I, Table 2) were diluted in blocking solution and incubated overnight at 4°C (F4/80 [Bio-Rad]: 10 μg/ml, aquaporin 9 [Battistone lab]: 0.5 μg/ml, V-ATPase B1 subunit [Battistone lab]: 0.3 μg/ml, cleaved-caspase-3 [Cell Signaling]: 1:100 dilution). After washing, corresponding secondary antibodies were applied for 1 h at RT according to manufactureŕs recommendations (Appendix 1, Table 2). Directly fluorochromeconjugated antibodies were applied for 1 h RT (anti-EpCam-AF594 [BioLegend]: 1 μg/ml). Developing spermatids were visualized by acrosome staining using PNA (FITC-conjugated, 1 μg/ml) applied for 30 min at RT), and nuclei were counterstained using DAPI (5 μg/ml). Specimens were mounted in Mowiol mounting media and imaged using a Leica Stellaris 5 confocal microscope. For quantification, at least 10 fields per regions were counted and averaged per biological replicate.

### Flow cytometry

Pericardial PBS infusion was performed prior to further processing, as described previously.^6^ Epididymal regions (proximal [segment 1–5] and distal regions [segment 6–10]) and testes were collected and transferred into RPMI medium supplemented with 10% FCS. To ensure sufficient cell yield, left and right epididymal fragments from the same individual were pooled to generate a single biological replicate. Tissues were dissociated by chopping followed by enzymatic digestion for 20 min at 37°C in RPMI containing 10% FCS, 1.5 mg/ml collagenase A and 60 U/ml DNase I. Digested fragments were aspirated through 22G needles and filtered through a 70 μm cell strainer. After centrifugation at 400xg for 10 min at 4°C, cells were resuspended in RPMI medium with 10% FCS. Non-specific Fc receptor binding was blocked using TrueStain FcX reagent (BioLegend). Cells were stained with antibodies listed in Appendix Table 2, following the gating strategy in Suppl. Fig. S1F for 30 min at RT in 100 μl of FACS buffer (RPMI medium containing 2 mM EDTA and 0.5% BSA). Controls included omission of the target antibody, incubation with a respective isotype control or fluorescence minus one (FMO) stainings under identical conditions. After antibody incubation, cells were washed twice in FACS buffer. Cell viability was assessed using DRAQ7 staining (1:1000 dilution) for 15 min at RT. Flow cytometry was performed using a BD FACSymphony S6. Data were analyzed using FlowJo v10.10.0 with the DownSampleV3 plugin for expert gating and high-dimensional analysis.

### Statistical analysis

Statistical analysis was performed using GraphPad Prism (version 10.1.2). Detailed information for statistical analysis of particular experiments are stated in the respective figure legend. Overall, a p-value < 0.05 was considered significant.

## RNA extraction, library preparation and mRNA sequencing (mRNA-seq)

RNA was extracted from testis, proximal (segment 1–5) and distal epididymis (segment 6–10) using the RNeasy Mini Kit (Qiagen) after bead-based tissue homogenization (Tissue homogenizer Retsch, 2.8 mm stainless steel beads). RNA purification was performed following manufactureŕs recommendation with an additional on-column DNase digestion using RNase-free DNase Set (Qiagen). For genome-wide gene expression analysis, RNA sequencing libraries from isolated mRNA were generated and sequenced by the Institute for Lung Health (ILH), Genomics and Bioinformatics at the Justus-Liebig-University (JLU) Giessen (Germany). 1000 ng of total RNA was used for polyadenylated mRNA selection, followed by cDNA sequencing library preparation utilizing the Illumina^®^ Stranded mRNA Prep Kit (Illumina) according to the manufacturer’s instructions. Library quality was assessed by capillary electrophoresis (4200 TapeStation, Agilent) and sequencing of cDNA libraries were performed on the Illumina NovaSeq 6000 platform, generating 50 bp paired-end reads.

Demultiplexing and subsequent FASTQ generation were performed with Illumina’s bcl2fastq (2.19.0.316). Primary read processing (i.e. quality control, filtering, trimming, read alignment and generation of gene specific count tables) was conducted using the nf-core^26^ RNA-seq v3.7 bioinformatics pipeline (NEXTFLOW version 23.04.03) in Docker mode with the *Mus musculus* mm10 reference genome and gene annotation from Illumina’s iGenome repository (https://support.illumina.com/sequencing/sequencing_software/igenome.html). Raw read count tables were imported into R (R Core Team, 2021) for down-stream processing. Normalization and identification of differentially expressed genes were performed using DESeq2 with default parameter. Gene set enrichment analysis (GSEA) was carried out with the *cluster-Profiler* and *fgsea* R packages using GO and KEGG annotations. Heatmaps were generated using the *complexHeatmap* package^27^. Barplots were generated using the *ggplot2* package. All analysis code is available upon request.

The steady-state transcriptional profiles of *Cx3cr1*^high^ macrophages were compared to (i) other macrophage subsets, and (ii) between proximal (IS, caput) and distal regions (corpus, cauda) by reanalyzing existing data,^6^ GSE208244)).

## Results

### Elaborating a mouse model to deplete intraepithelial CX3CR1^+^ macrophages within the epididymis

To investigate the role of intraepithelial macrophages in regulating epididymal immunity, we established a transgenic mouse model designed to predominantly target CX3CR1^hi^-expressing cells. This approach was based on previous findings showing that CX3CR1 is highly expressed by intraepithelial macrophages, with their abundance progressively declining from proximal to distal epididymal regions.^6^

Although interstitial CX3CR1^+^ macrophages and monocytes are also present in distal segments, these populations exhibit high turnover due to continuous recruitment from circulating monocytes.^6^ To account for this dynamic exchange and ensure effective depletion of more stably resident intraepithelial subsets (excluding short-living neutrophils and monocytes), we implemented a two-week interval between tamoxifen (Tam)-induced diphtheria toxin receptor (DTR) expression and subsequent diphtheria toxin (Dtx) administration for targeted depletion in *Cx3cr1*^CreER^*Rosa26*^iDTR/tdTomato^ mice (Fig. 1A). To visualize and confirm successful Tam-induced recombination, a tdTomato (Tom) reporter allele was also incorporated into the Rosa26 locus. Following this regimen, we observed a significant reduction of Tom^+^ cells in both proximal and distal epididymal regions. A milder but still significant decrease was also detected in the testis (Fig. 1B). Across all examined tissues, F4/80^+^CX3CR1^+^ cells accounted for almost all Tom^+^ cells, confirming specific targeting of CX3CR1^+^ macrophages (Fig. 1C). Administration of Tam or Dtx alone did not alter (i) other immune cell populations and (ii) the ratio of CX3CR1^+^F4/80^+^ cells within the CD45^+^ cells in either the epididymis or the testis (Suppl. Fig. S1A).

Confocal microscopy revealed that in proximal regions (initial segment, caput) Tom^+^ cells were predominantly intraepithelial/epithelia-associated macrophages (Fig. 1D). In distal regions (corpus, cauda), Tom + cells included both intraepithelial and a substantial number of interstitial macrophages (Fig. 1E). However, Dtx administration selectively reduced intraepithelial macrophages across all regions, while interstitial CX3CR1^+^ macrophages were only mildly affected (Fig. 1D,E), consistent with our strategy to preferentially deplete the more stable resident subsets.

High-dimensional analysis of CD45^+^ cells confirmed that tdTomato fluorescence was selectively restricted to CX3CR1^+^ macrophages, with no detectable expression in other investigated populations (including CX3CR1^−^ macrophages, monocytes, neutrophils, T cells or B cells) confirming the specific targeting of macrophages. This Tom^+^CX3CR1^+^ macrophage population was strongly diminished following Dtx administration (Fig. 1F,G, Suppl. Fig. S1B, S1C). No such changes occurred with Tam or Dtx treatment alone (Suppl. Fig. S1D).

Overall, the significant decrease in CD45^+^ cell numbers observed in both proximal and distal regions was primarily attributable to the loss of CX3CR1^+^ macrophages, while other immune cell populations remained quantitatively stable (Fig. 1H,I). Notably, the macrophage depletion was accompanied by a relative increase in neutrophils and T cells, suggesting a compensatory shift of pro-inflammatory immune cells in the local immune landscape, particularly within proximal regions of the epididymis (Suppl. Fig. S1E).

### Loss of CX3CR1^+^ macrophages results in focal epithelial damage and impaired sperm maturation within the epididymis

**Fig. 2.**
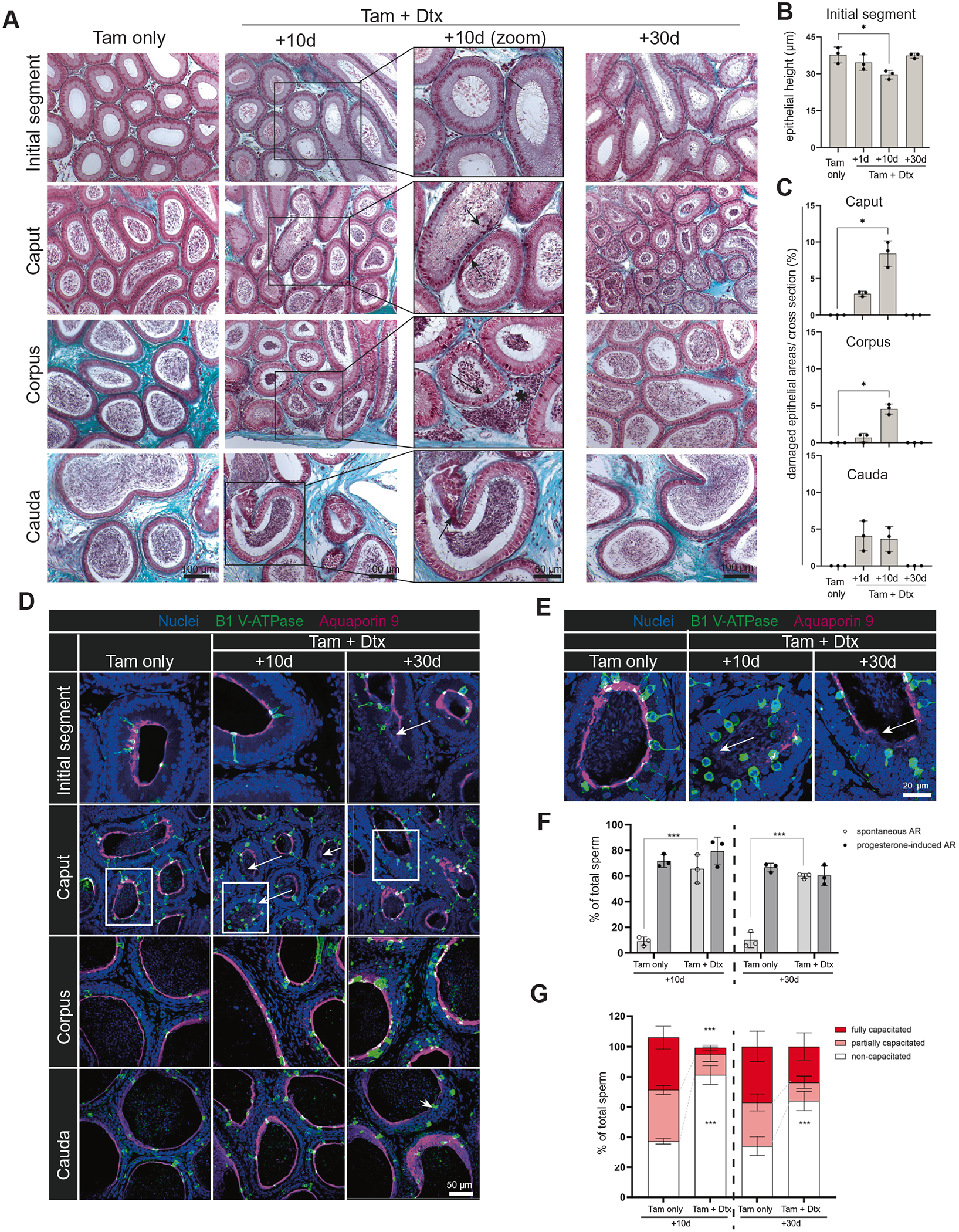
Focal epithelial damage and impaired sperm maturation following Dtx-mediated depletion of CX3CR1^+^ macrophages under physiological conditions. (A) Histological analysis of proximal (Initial segment/Caput) and distal (Corpus/ Cauda) regions at 10 and 30 days post-depletion. Masson Goldner trichrome staining. Arrows denote epithelial disruption, and asterisks indicate extravasated sperm. (B) Quantification of epithelial height in the initial segment at the indicated time points. Each data point represents one biological replicate (n = 3); 9–10 duct cross-sections (each containing 7–8 epithelial areas) were measured and averaged per sample. (C) Frequency of damaged epididymal duct cross-sections in caput, corpus and cauda at indicated time points post-depletion (n = 3). (D, E) Confocal microscopy of V-ATPase^+^ clear cells (green) and apical aquaporin 9 (purple) layers in IS, caput, corpus, cauda at 10 and 30 days post-depletion. Nuclei are stained with DAPI (blue). Arrows indicate disrupted aquaporin layers. Lined boxes highlight regions enlarged in (E). (F) Acrosome reaction (AR) in cauda-derived spermatozoa after in vitro capacitation with and without progesterone stimulation, assessed at 10 and 30 days post-depletion in Tam only and Tam + Dtx-treated mice (n = 3 per group). AR was evaluated using PNA staining (see representative images in Fig. S2D). (G) Assessment of sperm capacitation by tail protein tyrosine phosphorylation using 4G10 antibody. Sperm were classified as non-capacitated (4G10^−^), partially capacitated (spotted 4G10^+^), or fully capacitated (uniform 4G10^+^), and quantified by confocal microscopy (≥200 sperm per replicate, n = 3; representative patterns shown in Fig. S2d). Data are presented as mean ± SD. Statistical analysis was performed using Kruskal–Wallis test with Dunn’s post hoc correction (*p < 0.05, **p < 0.005, ***p < 0.001).

Although overall epididymal morphology appeared unaffected immediately after Dtx administration (Suppl. Fig. S2A), CX3CR1^+^ macrophage-depleted mice developed pronounced focal tissue damage peaking at 10 days after Dtx-mediated depletion, particularly in segments S2, S5/6 and S8 (Fig. 2A). Surprisingly, no overt epithelial damage was observed in the initial segment (Fig. 2A), despite this region being naturally the most densely populated with intraepithelial macrophages. Nonetheless, a significant reduction in epithelial height indicated impaired secretory function (Fig. 2B). In more distal regions, up to 10% of epithelial areas exhibited structural damage, characterized by loss of epithelial integrity, pyknotic epithelial cells, exfoliation of epithelial cells, accumulation of immature germ cells in the epididymal lumen and extravasation of sperm into the interstitial space (Fig. 2A, Fig. 2C). The loss of epithelial integrity was further confirmed by immunofluorescence stainings for Aquaporin 9 (AQP9) and EPCAM, both of which showed marked epithelial discontinuities (Fig. 2D and Suppl. Fig. S2B, respectively). Intriguingly, while the gross morphology appeared to recover towards 30 days after depletion (Fig. 2A), both the apical AQP9 layer as well as EPCAM integrity exhibited persistent impairments of the epithelial barrier not observed with Tam or Dtx treatment alone (Fig. 2E and Suppl. Fig. S2B,C). Notably, there was no increase in apoptotic cell numbers (cleaved caspase 3 staining, Suppl. Fig. S2B) and no detectable change in clear cells numbers (Fig. 2D). Tam or Dtx treatment alone did not affect epithelial integrity (Suppl. Fig. S2C).

To assess functional consequences for sperm maturation, we analyzed sperm isolated from the cauda region. Sperm from CX3CR1^+^ macrophage-depleted mice exhibited a significantly elevated rates of spontaneous acrosome reactions (AR), reaching levels comparable to those induced by progesterone stimulation (Fig. 2F, Suppl. Fig. S2D). Regarding capacitation, a prerequisite for acquiring hypermotility within the female reproductive tract, significantly fewer sperm displayed tyrosine phosphorylation (a molecular hallmark of successful capacitation) following *in vitro* stimulation (Fig. 2G, Suppl. Fig. S2D). Mice depleted of CX3CR1 macrophages did not develop anti-sperm antibodies after 30 days under physiological conditions (Suppl. Fig. S2E), consistent with the absence of inflammatory responses during the 30 day course after Dtx-mediated depletion, suggesting that the depletion of CX3CR1 macrophages alone is not sufficient for the development of anti-sperm autoimmunity.

Collectively, these findings indicate that the loss of epithelial integrity following CX3CR1^+^ macrophage depletion directly compromises sperm maturation, likely due to disrupted epithelial function and subsequent alterations in the luminal microenvironment required for proper sperm development, as opposed to anti-sperm responses.

### Influence of the CX3CR1^+^ macrophage depletion on the testicular macrophage pool

**Fig. 3.**
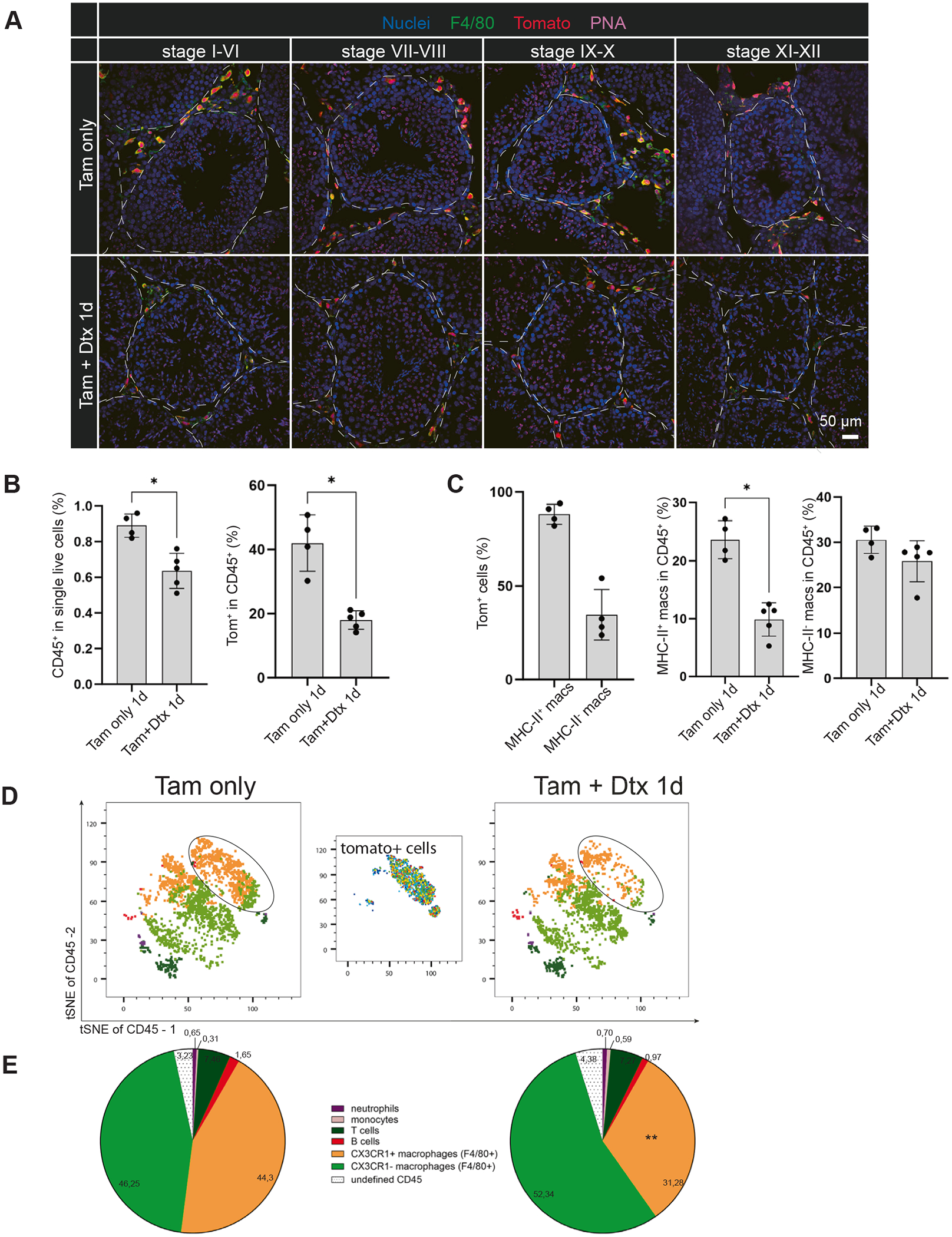
Impact of Dtx-mediated depletion on testicular macrophages at 1 day after depletion. (A) Confocal microscopy images showing the distribution of Tom^+^ cells (red) in the testis, aligned with distinct stages of spermatogenesis. Macrophages were co-stained with an antibody against F4/80 (red) and nuclei were stained using DAPI (blue) and developing acrosomes were visualized with PNA (purple) to distinguish spermatogenic stages. (B) Relative abundance of CD45^+^ cells among live single cells, and proportion of Tom^+^ cells within the CD45^+^ compartment, one day after depletion in Tam only and Tam + Dtx-treated (+1d) mice (n = 4–5 from two independent experiments). (C) Proportion of Tom^+^ cells within MHC-II^+^ and MHC-II^−^ testicular macrophage subsets, and relative ratios of MHC-II^+^ and MHC-II^−^ macrophages within total CD45^+^ cells, in Tam only and Tam + Dtx-treated mice (+1d) after depletion (n = 4–5). (D) t-SNE plots (opt-SNE with iterations: 500, perplexity: 20) of testicular CD45^+^ populations from representative Tam only and Tam + Dtx-treated mice, one day post depletion. CD45^+^ cells were downsampled, gated as shown in Fig. S1F and overlaid onto the t-SNE plots. Tomato + cells were separately gated and plotted on the t-SNE coordinates. Circles indicate differences in CX3CR1^+^ macrophages between Tam only and Tam + Dtx-treated mice. (E) Pie charts showing the relative composition of immune cell populations within the testicular CD45^+^ compartment in Tam only and Tam + Dtx-treated mice, one day post-depletion. Cell populations were defined using the gating strategy in Fig. Fig. S1F and are color-matched to the t-SNE plots in (D). Mean values of indicated cell types are indicated in numbers [% in CD45^+^]. Statistical analysis was performed using the Kruskal–Wallis test with Dunn’s post hoc correction (*p < 0.05, **p < 0.005, ***p < 0.001). Data are presented as mean ± SD from two independent experiments. Statistical analysis was performed using the Mann–Whitney *U* test (*p < 0.05, **p < 0.005, ***p < 0.001).

Given that testicular macrophages also express CX3CR1^28^ and CX3CR1^+^ macrophage-depleted mice exhibit increased numbers of immature germ cells in the epididymal lumen, we next examined how the depletion strategy affected the testicular macrophage pool. Overall, no specific macrophage subset appeared to be preferentially tdTomato-labeled or selectively depleted, even when considering the spatial distribution and density of macrophages in relation to the spermatogenic stages, suggesting the transgenic construct targeted both interstitial and peritubular macrophages (Fig. 3A). Among all immune cells (CD45^+^), overall numbers were reduced by approximately 20%, with around 40% of immune cells showing tdTomato labeling (Fig. 3B). Although Dtx-mediated depletion caused milder reductions in the testis compared to the epididymis (Fig. 1B), the number of CX3CR1^+^ macrophages per mg tissue was significantly decreased 1 day after Dtx-treatment, followed by recovery at 10 and 30 days post-depletion, similar to the pattern observed in the epididymis (Suppl. Fig. S1B, Suppl. Fig. S3A). When distinguishing between MHC-II^+^ and MHC-II^−^ macrophages, the MHC-II^+^ subset was predominantly tdTomato-labeled and thus selectively depleted by Dtx, whereas MHC-II^−^ macrophages remained largely unaffected (Fig. 3C, Suppl. Fig. S3B). High-dimensional analysis, confirmed that tdTomato expression was restricted to CX3CR1^+^ macrophages, which were specifically targeted by the depletion strategy (Fig. 3D). Other immune cell populations remained stable and were unaffected by either combined treatment or by Tam or Dtx alone (Fig. 3E, Suppl. Fig. S3C).

### Loss of CX3CR1^+^ macrophages results in impaired spermatogenesis and steroidogenesis

Previous studies employing a related model that targets all macrophages, including those originating from CX3CR1^+^ progenitors, demonstrated disrupted spermatogonial differentiation.^15^ Building on these findings, we investigated how selective depletion of CX3CR1^+^ macrophages affects spermatogenesis in our model.

**Fig. 4.**
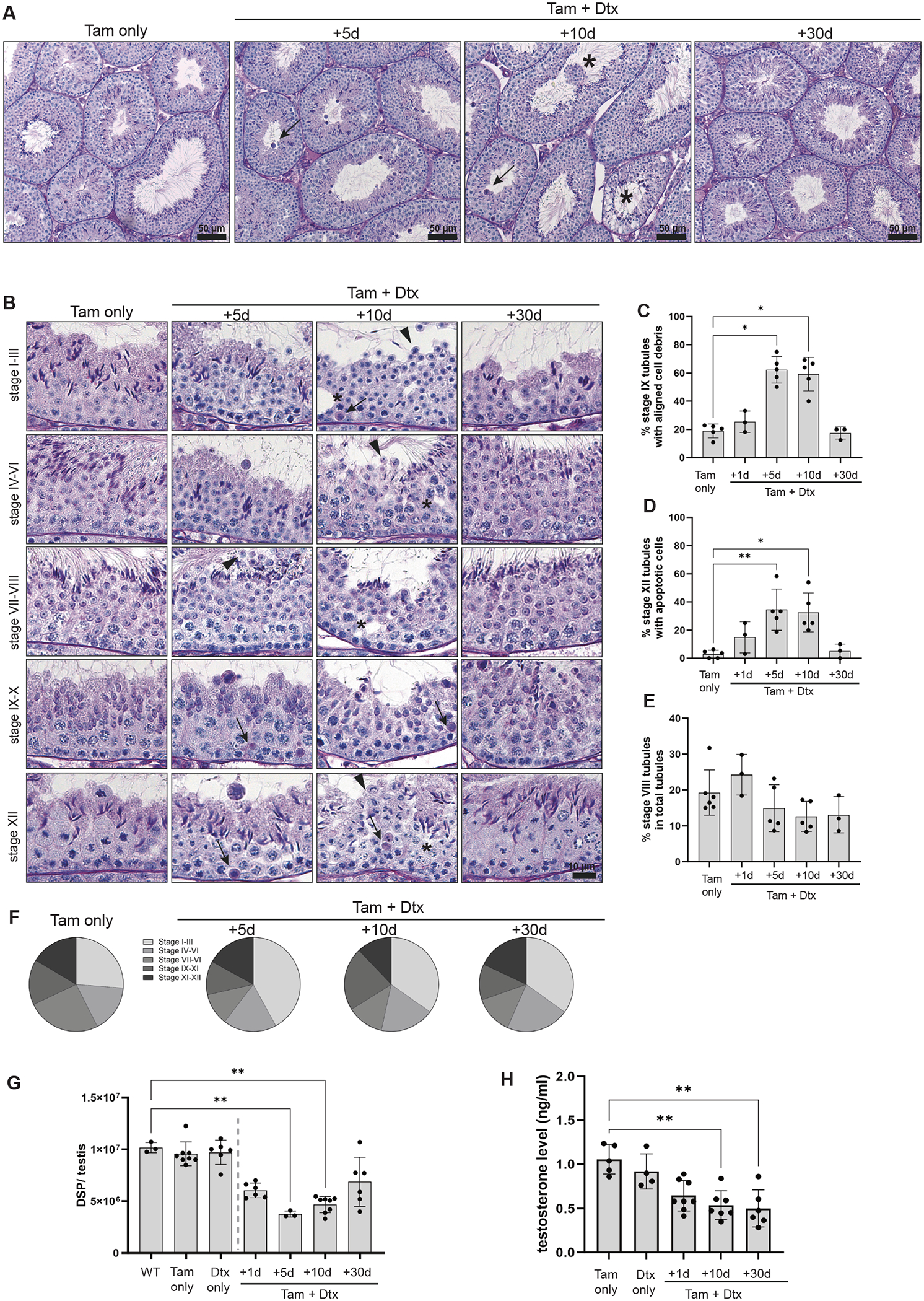
Spermatogenic disruption in the testis following macrophage depletion under physiological conditions. (A) Gross histological analysis of testis from Tam only and Tam + Dtx-treated mice at 5, 10 and 30 days post-depletion. Periodic acid-Schiff (PAS)-staining. Arrows indicate round apoptotic cells and asterisks denote focal damage in seminiferous tubules. Periodic acid Schif́fs staining. (B) Higher-magnification images highlighting histological abnormalities at specific spermatogenic stages in Tam only and Tam + Dtx-treated mice. Arrowheads indicate exfoliating round spermatids. Arrows mark apoptotic cells and asterisks denote structural lesions in the seminiferous epithelium. (C) Proportion of seminiferous tubules with apically aligned cell debris at stage IX (post-spermiation). Each data point represents one biological replicate (n = 3–5). (D) Proportion of seminiferous tubules showing apoptotic germ cells during stage XII (second meiotic division, each dot represents one biological replicate, n = 3–5). (E) Frequency of stage VIII tubules (spermiation stage) relative to total tubules (n = 3–5). (F) Distribution of spermatogenic stages across all tubules, shown as pie charts for Tam only and Tam + Dtx-treated groups (mean of n = 3–5). (G) Daily sperm production per testis in C57BL/6J wild type, Tam only, Dtx only and Tam + Dtx-treated mice at indicated time points. Each dot represents one biological replicate (n = 3–8 from ≥ 2 independent experiments). (H) Serum testosterone levels from Tam only, Dtx only and Tam + Dtx-treated mice at indicated time points (n = 5–8 from two independent experiments). Data are presented as mean ± SD. Statistical analysis was performed using the Kruskal–Wallis test with Dunn’s post hoc correction (*p < 0.05, **p < 0.005, ***p < 0.001).

Detailed analysis revealed distinct alterations across spermatogenic stages (Fig. 4B). Early haploid germ cells (spermatids) were frequently detached from the seminiferous epithelium at stages I–III and IV–VI, and elongated (advanced) spermatids were markedly reduced in macrophage-depleted mice, indicating reduced spermatogenic output. At stage IX, immediately following sperm release, apoptotic cells and debris accumulated at the apical seminiferous epithelium (Fig. 4C), as quantified by cleaved caspase–3 immunostaining (representative image in Suppl. Fig. S4E). The reduction in elongated (advanced) spermatids was accompanied by increased apoptotic spermatocytes at stage XII – the stage of second meiotic division – suggesting defective germ cell differentiation and impaired meiotic progression (Fig. 4D).

Although the proportion of tubules at the sperm release stage (stage VIII) showed only a non-significant downward trend (Fig. 4E), all spermatogenic stages were still present in macrophage-depleted mice at all time points examined (Fig. 4F), indicating that spermatogenesis was impaired but not entirely abrogated. By 30 days post-depletion, histopathological changes had reverted to Tam-treated control levels (Fig. 4A–F), suggesting that the observed effects were reversible under steady-state conditions.

No alterations in gross morphology or spermatogenic staging were observed in mice treated with Tam or Dtx alone (Suppl. Fig. S4A,B).

Spermatogenic impairment was reflected in a significant reduction in daily sperm production per testis in macrophage-depleted mice compared to non-induced littermates and single treatment controls (Fig. 4G). Despite the quantitative impairment in spermatogenesis, overall total testis weight remained largely unaffected until 30 days after depletion (Suppl. Fig. S4C) – at least in relation to total body weight (Suppl. Fig. S4D) suggesting a mild rather than severe disruption of overall spermatogenic function.

Notably, steroidogenesis was also impaired, as evidenced by significantly reduced serum testosterone levels (Fig. 4H). This decrease had functional consequences, reflected by reduced seminal vesicle size and weight – an organ highly sensitive to testosterone fluctuations (Suppl. Fig. S4F). Notably, despite significantly reduced testosterone levels, circulating luteinizing hormone (LH) concentrations remained unchanged (Suppl. Fig. S4G), indicating that macrophage depletion impairs testosterone production at the testicular level rather than throught disruption of the hypothalamic-pituitary axis.

### Replenished macrophages are distinct from the original population

Given the observed recovery of histopathological alterations in both the epididymis and testis, we investigated by flow cytometry and immunostainings if and how the macrophage pool recovers following Dtx-mediated depletion.

**Fig. 5.**
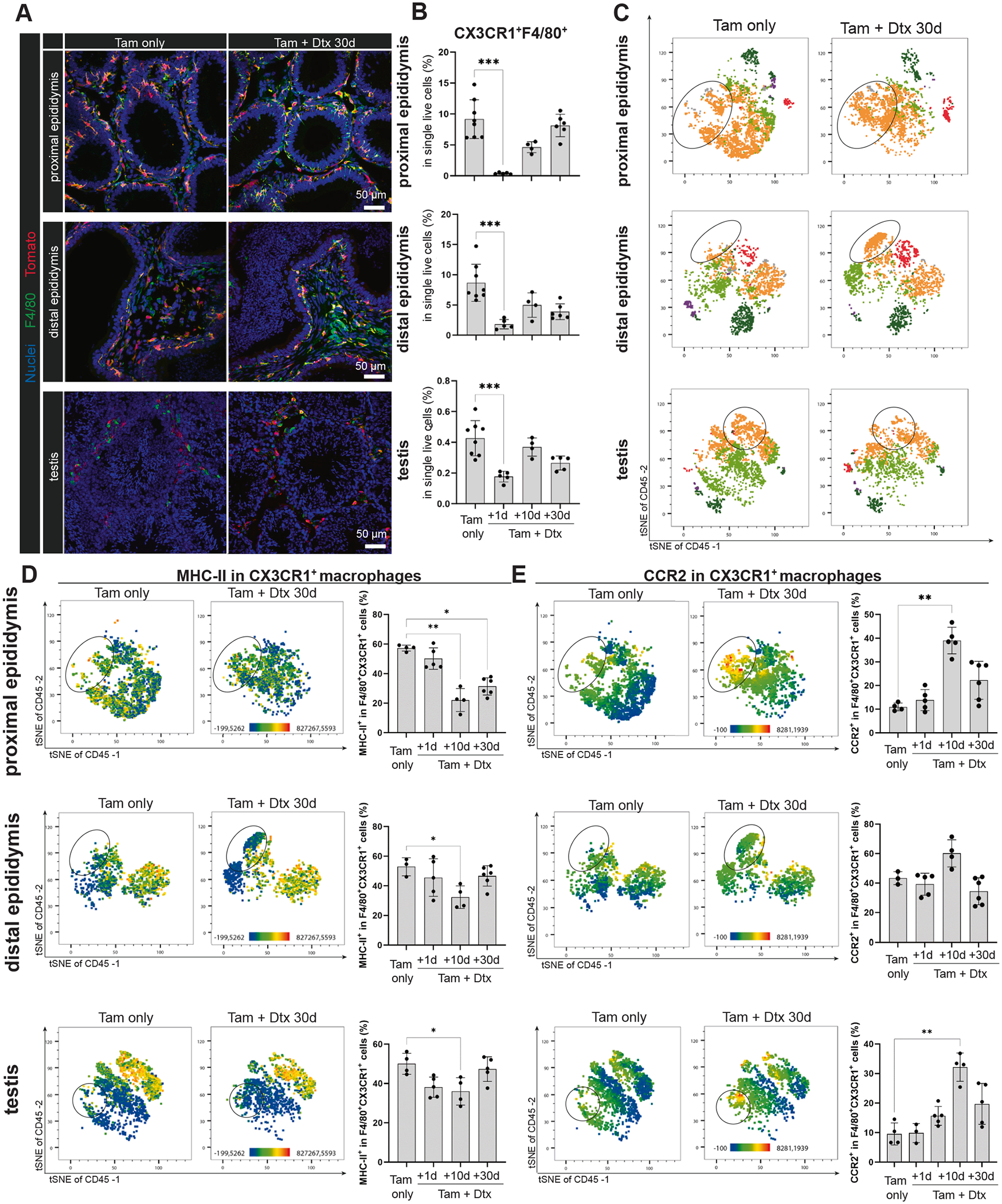
Replenishment of testicular and epididymal macrophages following depletion. (A) Confocal microscopy images showing Tom^+^ cells within the proximal and distal regions and the testis in Tam only and Tam + Dtx-treated mice, 30 days post-depletion. (B) Proportion of CX3CR1^+^F4/80^+^ macrophages among single live cells at indicated time points post depletion (n = 4–8 from two independent experiments). (C) t-SNE plots (opt-SNE with iterations: 500, perplexity: 20) of proximal and distal epididymis and testis from representative Tam only and Tam + Dtx-treated mice, 30 days post depletion. CD45^+^ cells were downsampled, gated as shown in Fig. S1F and overlaid onto the t-SNE plots. Circles indicate differences in CX3CR1^+^ macrophages between Tam only and Tam + Dtx-treated mice. (D, E) t-SNE plots from C selected for all macrophages with overlaid MHC-II (D) and CCR2 (E) expression and respective proportions of MHC-II^+^ and CCR2^+^ macrophages quantified by flow cytometry at the indicated time points (n = 4–8). Circles indicate differences in CX3CR1^+^ macrophages between Tam only and Tam + Dtx-treated mice. Data are presented as mean ± SD. Statistical analysis was performed using the Kruskal–Wallis test with Dunn’s post hoc correction (*p < 0.05, **p < 0.005, ***p < 0.001).

Previously depleted niches were repopulated by macrophages that, at the phenotypic level, resembled those in Tam-only control mice in both proximal and distal epididymal regions as well as the testis 30 days after depletion (Fig. 5A, Suppl. Fig. S5A). The proportion of F4/80^+^CX3CR1^+^ cells also returned to baseline levels by 30 days post depletion (Fig. 5B). Although the macrophage pool quantitatively recovered, high-dimensional analysis and dimensionality reduction based on key subset and activation markers (CX3CR1, CCR2, MHC-II, CD163, and other investigated immune cell markers) revealed that newly recruited macrophages were differentially situated in immune cell clustering when comparing Tam-only and Tam + Dtx-treated mice 30 days after Dtx administration (Fig. 5C).We further used high-dimensional flow cytometry to assess differential marker expression of CX3CR1^+^ macrophages in Tam + Dtx and Tam only treated mice at 30 days after depletion. In both the epididymis and testis, MHC-II^+^ macrophages were not only disproportionally affected by the depletion, but also exhibited significantly reduced MHC-II expression after repopulation – particularly in proximal epididymal regions – suggesting diminished antigen-presenting capacity (Fig. 5D). Additionally, a fraction of CX3CR1^+^ macrophages in Tam + Dtx treated mice displayed elevated CCR2 expression (Fig. 5E), indicating a partial contribution of monocyte-derived cells to the repopulating macrophage pool. Under normal steady-state conditions, this feature is restricted to the cauda region within the epdidymis.^6^ Notably, following depletion, we observed a transient increase in macrophage proliferation, at least within the epididymis (Fig. S5B), as indicated by a higher proportion of KI67 + cells within remaining interstitial macrophages (Suppl. Fig. S5B). During the recovery phase, the proportion of CCR2^+^ macrophages increased temporarily (Fig. 5E). Over the 30-day observation period, the fraction of Tom + macrophages showed a gradual but relatively mild decrease in all investigated organs (Suppl. Fig. S5C).

The most pronounced differences between repopulated and resident macrophages occurred in proximal epididymal regions, where intraepithelial CX3CR1^+^ macrophages are naturally most abundant and closely associated with the epididymal luminal content.

### Depletion and subsequent dysfunctional repopulation of the CX3CR1^+^ macrophages niche could both contribute to an exacerbated epididymal immune response to bacterial infection

**Fig. 6.**
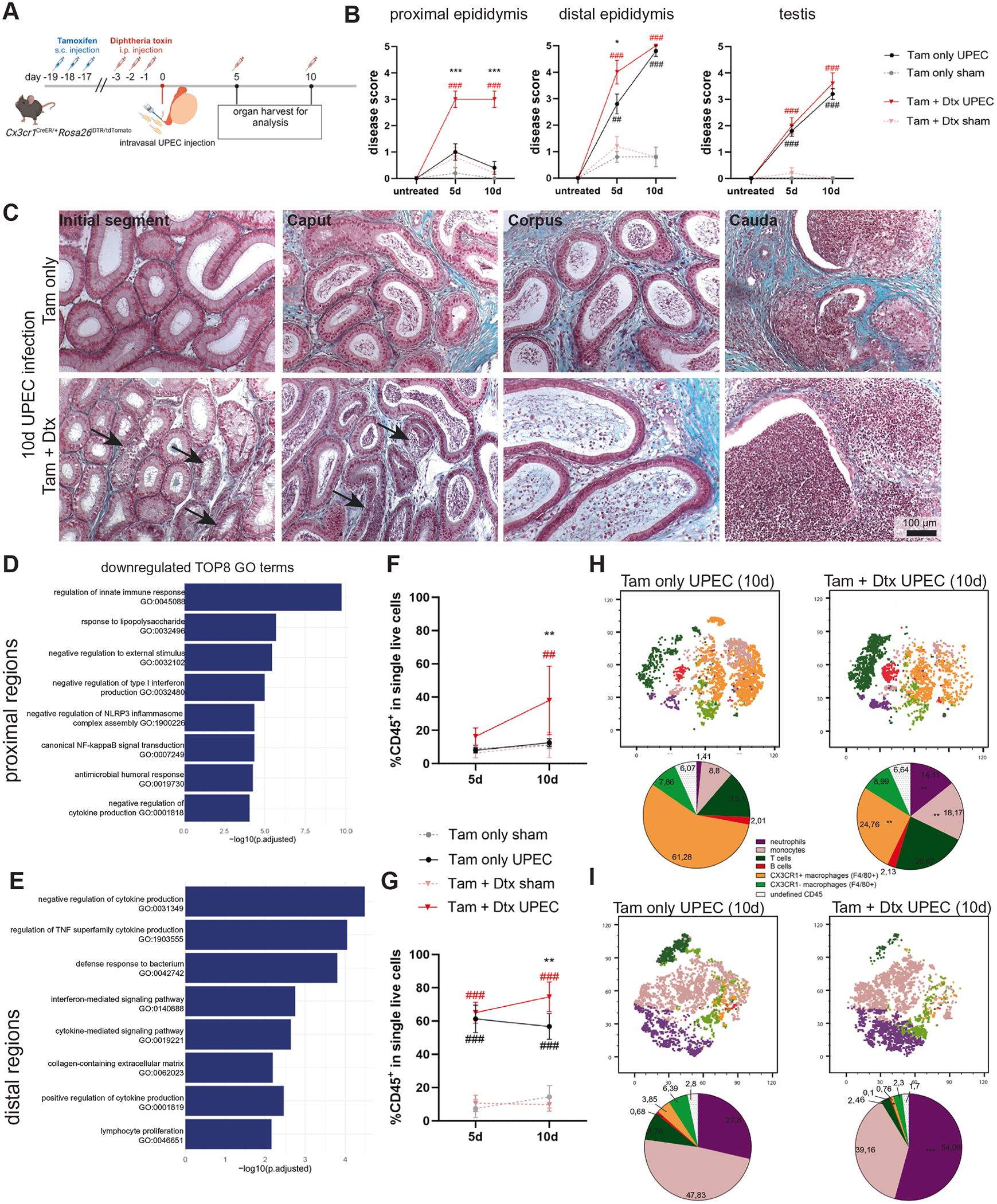
Impact of CX3CR1^+^ macrophage depletion on immune responsiveness during acute bacterial epididymitis. (A) Experimental protocol outlining sequential Tam and Dtx treatment in *Cx3cr1*^CreER/+^*Rosa26*^iDTR/tdTomato^ mice, followed by intravasal injection of uropathogenic *Escherichia coli* (UPEC) to induce acute bacterial epididymitis. (B) Disease scores in proximal and distal epididymis, and testis at 5 and 10 days after sham or UPEC infection in Tam only and Tam + Dtx-treated mice (n = 5 per group from two independent experiments). Scoring was based on established parameters (see Methods). Asterisks indicate the Dtx effect in UPEC-infected mice, red # indicate UPEC effect in Tam + Dtx treated mice and black # indicate UPEC effects in Tam only treated mice. Data are presented as mean ± SD. Statistical analysis was performed using ANOVA (*p < 0.05, **p < 0.005, ***p < 0.001). (C) Representative histological images from Tam only and Tam + Dtx-treated mice, 10 days post-UPEC infection, showing epithelial damage across all four epididymal regions. Arrows highlight areas of pronounced damage in the IS and caput of Tam + Dtx-treated mice. Masson-Goldner-Trichrome-Staining. (D, E) Gene set enrichment analysis (GSEA) based on differentially expressed genes (DEGs) between Tam only and Tam + Dtx-treated mice at 5 days after UPEC infection. Top eight downregulated gene ontology (GO-term shown; (n = 3). (F, G) Relative abundance of CD45^+^ cells among single live cells at 5 and 10 days after UPEC infection in Tam only and Tam + Dtx-treated mice. Asterisks indicate the Dtx effect in UPEC-infected mice, red # indicate UPEC effect in Tam + Dtx treated mice and black # indicate UPEC effects in Tam only treated mice. (H, I) t-SNE plots (opt-SNE with iterations: 1000, perplexity: 30) of CD45^+^ populations within proximal (H) and distal (I) epididymis from representative Tam only and Tam + Dtx-treated mice, 10 days after UPEC infection. CD45^+^ cells were downsampled, gated as shown in Fig. S1F and overlaid onto the t-SNE plots. Respective pie charts showing the relative composition of CD45^+^ immune subsets, defined by the gating strategy in Fig. S1F and color-matched to the t-SNE plots. Mean values of indicated cell types are indicated in numbers [% in CD45^+^]. Statistical analysis was performed using the Kruskal–Wallis test with Dunn’s post hoc correction (*p < 0.05, **p < 0.005, ***p < 0.001).

Based on their dense intraepithelial distribution and unique transcriptional profile (characterized by a predominantly homeostatic signature compared to other epididymal macrophage subsets (Suppl. Fig. S6A), yet simultaneously adapted (homeostatic in the proximal epididymis and enriched for defense factors in distal regions, Suppl. Fig. S6B) to the specific needs of the respective regions) —we hypothesized that intraepithelial CX3CR1^+^ macrophages act as key regulators, tipping the balance in region-specific immune responses toward intraluminal pathogens. To test this, we induced an acute bacterial epididymitis by intravasal injection of uropathogenic *Escherichia coli* (UPEC) following macrophage depletion (Fig. 6A).

At day 10 after infection, mice depleted of CX3CR1^+^ intraepithelial macrophages exhibited markedly heightened immune response, particularly in proximal epididymal regions that normally remain largely unresponsive when infected with UPEC under control conditions (Fig. 6B). The exacerbated immune response was characterized by severe epithelial damage, exfoliation of epithelial cells, interstitial immune infiltrates and extravasation of spermatozoa (Fig. 6C). Moreover, inflammation also arose earlier across all epididymal regions (Suppl. Fig. S6C) with significantly more severe tissue damage, especially in the cauda region, detected by day 5 after infection. In contrast, testicular responses were comparable between groups (Fig. 6B).

Transcriptional profiling revealed a pronounced downregulation of genes involved in innate immune regulation, lipopolysaccharide responses, negative regulation of type I interferon production, NLRP3 inflammasome activation and responses to external stimuli in proximal regions in CX3CR1^+^ macrophage-depleted mice. These findings suggest that intraepithelial macrophages normally restrain excessive proinflammatory responses following bacterial infection and thereby preserve tissue integrity (Fig. 6D, Suppl. Fig. S6D). In distal regions, genes associated with cytokine regulation (e.g. TNFα production), antibacterial defense, and collagen-containing extracellular matrix production were downregulated in CX3CR1^+^ macrophage-depleted mice, indicating a dysregulation of inflammatory processes (Fig. 6E, Suppl. Fig. S6D), potentially contributing to the observed earlier onset of severe tissue-damaging inflammation.

Consistent with these transcriptional changes seen at day 5 post-infection, significant differences in the magnitude of immune cell infiltration were observed at day 10 in both proximal and distal epididymal regions (Fig. 6F,G, respectively). Neutrophils were particularly increased in both regions (Fig. 6H,I), while proximal regions additionally showed a significantly amplified ratio of T cells, indicating an activation of adaptive immunity (Suppl. Fig. S7D). Interestingly, CX3CR1^+^ macrophages repopulated more rapidly in proximal epididymal regions of UPEC-infected mice and overlap phenotypically with monocytes (Fig. 6H,I, Suppl. Fig, S7D), suggesting that infection drives enhanced monocyte recruitment and repopulation of the depleted niches.

Notably, the heightened inflammatory response observed across all epididymal regions was not due to impaired bacterial clearance, as bacterial loads remained comparable between macrophage-depleted and Tam-only control mice at 10 days post-infection (Suppl. Fig. S7E).

Within the testis, no significant differences in immune response were observed, as indicated by comparable magnitudes of histopathological alterations between macrophage-depleted and control mice (Suppl. Fig. S8A,B). Only minor transcriptional changes occurred, including upregulation of genes involved in extracellular matrix remodeling in CX3CR1^+^ macrophage-depleted mice. Conversely, downregulated processes related to sister chromatid segregation, regulation of chromosome organization, and microtubule dynamics likely reflect the impact of macrophage depletion on germ cell differentiation effects previously noted under uninfected conditions and now potentially exacerbated in the context of inflammation (Suppl. Fig. S8C). Consistent with the histopathology, both macrophage-depleted and control groups exhibited only mild immune cell infiltration following UPEC infection, with a trend toward increased neutrophil and T cell accumulation in macrophage-depleted mice (Suppl. Fig. S8D,E).

Overall, these findings indicate that CX3CR1^+^ macrophages are critical regulators of immune responses in the epididymis, but to a lesser extent in the testis. In the epididymis they likely serve as key drivers of a tightly controlled immune environment maintaining the delicate balance between effective pathogen defense and preservation of tissue integrity.

## Discussion

Immune regulation and tissue integrity are essential for the specialized microenvironments that support male germ cell development, sperm maturation and fertility. In line with previous studies,^10^ our data show that epithelium-associated CX3CR1^+^ macrophages in the epididymis are highly regionally adapted and central to tissue homeostasis in both steady-state and pathological contexts. Although phenotypically distinct from testicular macrophages,^5^ they share the overarching function of supporting key physiological processes. Targeted depletion of CX3CR1^+^-macrophages disrupted testicular germ cell development and epididymal sperm maturation, underscoring their essential role.

Unlike the immune-privileged testis, the epididymis is not a primarily immune-privileged site. Its immune regulation relies heavily on the epithelial integrity (maintained by the blood-epididymis barrier formed by apical junctional complexes between principal cells) and a unique epithelial cell composition.^29,30^ Intraepithelial mononuclear phagocytes and epithelial cells, particularly clear cells, are strategically positioned and interact to shape an immune-protective environment in which sperm mature and are stored, shielded from autoimmune responses and pathogens.^6,31^ Recent findings suggest that these interactions are region-specific.^32^ Although clear cell abundance and morphology remained unchanged, their function is likely impaired following the selective depletion of intraepithelial macrophages, which may contribute to the observed defects in sperm maturation. Indeed, previous studies have suggested that macrophages stimulate clear cell activity (e.g. by the chemokine RANTES) required for luminal acidification under steady-state conditions.^14^

Evidence from efferent duct ligation (EDL) further supports the role of epididymal macrophages in maintaining epithelial homeostasis. EDL triggers apoptosis of epithelial cells by blocking luminal testicular factors. Damaged cells are cleared by efferocytosis,^33^ a macrophage-driven process that prevents secondary inflammation and promotes epithelial repair. Moreover, recent studies evidenced that F4/80^+^ macrophages are involved in phagocytosis of extravsated sperm following epithelial damage under steady-state conditions.^34^ Together these findings highlight important epithelial cell – macrophage circuits in immunoregulation and maintenance of epididymal tissue homeostasis, functions likely impaired by macrophage depletion and contributing to epithelial disintegration and defective sperm maturation.

The initial trigger for observed long-term changes is unequivocally the depletion of the resident macrophage population, which creates the niche instability that precedes repopulation. However, our data also show that the replenished macrophage pool is phenotypically distinct. Therefore, the persistent impairments in sperm maturation under steady-state as well as the exacerbated immune response to infection – particularly within proximal regions – could be driven not only by the absence of the original regulatory/homeostatic cells but also by the proinflammatory activity of newly recruited macrophages. This interpretation aligns with recent findings demonstrating that repopulating macrophages exhibit functional and transcriptional profiles distinct from original tissue-resident macrophages, often skewed toward a more proinflammatory phenotype.^35,36^

Of note, macrophage loss caused focal epithelial damage and sperm extravasation without inducing overt inflammation or anti-sperm antibody production under non-infected conditions. Thus, intraepithelial macrophages appear not to be the primary drivers of peripheral tolerance to sperm, a function more typically attributed to dendritic cells^37^ and regulatory T cells.^34,38^

Maintaining epithelial integrity—and thus strict compartmentalization of developing sperm—, however, is crucial to prevent anti-sperm immunity, especially under pathological conditions. In wild type naive mice, the morphology of proximal epididymal regions remains mostly unaffected upon bacterial infection, while distal regions, particularly the cauda, undergo severe tissue remodeling.^6–8^ Despite pathogen clearance, chronic inflammation often persists, mirroring clinical epididymitis where up to 40% of patients experience lasting sub-/infertility and develop anti-sperm antibodies.^2,3^ Our previous and current studies in experimental mice show that loss of epithelial integrity in the cauda is a critical event, resulting in the leakage of luminal content consisting of millions of sperm with UPEC into the immunologically active interstitial space^6^ (Pleuger unpublished observations). This exposure may trigger immune cross-reactivity, as pathogens like UPEC can act as adjuvants initiating an immunological cascade that targets other antigens as well.^39,40^

Our findings demonstrate that intraepithelial macrophages influence the onset of inflammation in a region-specific manner and are critical for modulating the course of disease at its initiation, particularly within proximal regions. In doing so, intraepithelial macrophages act in concert with other epithelial cells, including clear cells that were previously demonstrated to actively contribute to the mucosal immunity of the epididymis.^31,41^ Among the downregulated genes in proximal epididymal regions of UPEC-infected macrophage-deficient mice, we identified *Cxcl10* – a chemokine with known intrinsic antimicrobial activity against gram-negative bacteria^42^ and immunomodulatory effects via CXCR3 signaling.^43^ Notably *Cxcl10* is expressed by clear cells at steady-state in the caput epididymidis, and is further induced upon infection throughout the epididymis.^31^ Defining the macrophage-mediated induction/regulation of CXCL10 production in clear cells as well as targeting clear cell–derived CXCL10 and modifying the CXCL10-CXCR3 could provide insights into its role in disease progression and its potential as a prognostic and therapeutic target in translational settings.

In contrast, the macrophage-depleted testis did not show a significantly altered disease course compared to controls. This suggests that partial depletion of CX3CR1^+^ resident macrophages is insufficient to alter the testicular immune response – at least in the context of ascending bacterial infections that primarily affect the epididymis. Notably, even under wild-type conditions, testicular and epididymal immune responses differ, and inflammation-induced tissue damage in the testis is reversible.^8^ This likely reflects the immune-privileged status of the testis, where distinct regulatory mechanisms operate compared to the mucosal surface of the epididymis.^44^

Nevertheless, under physiological conditions, we observed clear impairments in both spermatogenesis and steroidogenesis coinciding with the partial loss of testicular macrophages underscoring the sensitivity of testicular function to even partial disruption of macrophage populations and emphasizing the essential role of CX3CR1^+^ macrophages in maintaining tissue homeostasis beyond overt inflammatory responses. Previous studies demonstrated that CX3CR1^+^ progenitors give rise to both interstitial and peritubular macrophages within the testis.^28^ In our model, MHC-II^+^ macrophages were predominantly affected, suggesting that mainly, but not exclusively, peritubular macrophages were targeted (though our immunolocalization studies demonstrated a reduction of both peritubular and interstitially located macrophages). Further marker-based stratification of macrophage subsets would be required but was beyond the scope of this study.

Consistent with our observations of spermatogenic impairment, previous studies demonstrated that resident macrophages regulate spermatogonial activity indirectly (by interacting with Leydig cells^45,46^ as well as directly through CSF1 and retinoic acid signaling.^15,45,46^ For the latter, peritubular macrophage-produced CSF1 targets CSF1R-expressing undifferentiated spermatogonia, facilitated by the close physical association between undifferentiated spermatogonia and peritubular macrophages which are separated only by thin cellular protrusions of the myoid peritubular cells.^15,47^

A related depletion model using *Cx3cr1*^Cre^ (instead of *Cx3cr1*^CreER^), which induced DTX expression in CX3CR1^+^ cells including also other CX3CR1-expressing cells throughout lifetime (including additional immune cell populations) resulted in a more extensive macrophage loss. Minor differences in depletion severity may reflect the tamoxifen-induced DTR expression used in our model, which was designed to selectively target monocyte-independent CX3CR1^+^ macrophages, particularly in the epididymis. Nevertheless, it showed similar phenotypic effects (attributed to disrupted macrophage-spermagonia interactions), and macrophage recovery in the testis.^15^ Of note, another study that employed CSF1 antibody-mediated macrophage depletion did not report gross morphological alterations or spermatogenic impairments in the adult testis.^48^ Although in our model spermatogenic stages were disrupted, spermatogenesis recovered in parallel with macrophage repopulation, suggesting that testicular function depends on a balanced immune homeostasis.

In both organs, the macrophage niches were replenished over time; however, the phenotype was not fully restored even 30 days after depletion. An increased proportion of CCR2^+^ cells suggests that monocytes at least partially contributed to niche repopulation, following an initial phase of proliferation of remaining local macrophage. This contrasts with the steady-state in wild-type mice, where testicular and proximal epididymal macrophages are largely maintained by self-renewal, and only distal epididymal regions undergo continuous replenishment by circulating monocytes.^5,6^ It is plausible that tissue stress or damage triggers the release of yet unidentified signals that recruit monocytes to repopulate the resident macrophage pool as observed in other tissues.^49,50^ However, our immunostaining and flow cytometry data provide rather supportive but not conclusive evidence that monocyte may partially participate to macrophage recovery as definite discrimination would require future studies using dedicated transgenic fate-mapping or parabiosis approaches.

Of note, although CX3CR1 is an effective driver for targeting macrophages within male reproductive organs (most efficiently within the epididymis), it is not entirely cell-type- or organ-type-specific. Consequently, CX3CR1^+^ macrophages in other tissues may also be affected, potentially influencing reproductive function systemically. This is particularly relevant for brain-resident macrophages, which express high levels of CX3CR1 and are long-lived, making them susceptible to this depletion strategy. Both microglia and other resident macrophage subsets reside within the hypothalamus and pituitary gland, respectively and are known to regulate local tissue homeostasis in both organs^51–53^ which can in turn affect systemic hormonal balance via the hypothalmus-pituitary gland-axis (HPG). In particular, depletion of pituitary macrophages has been shown to profoundly alter pituitary hormone levels, including reduced luteinizing hormone (LH,^53^ which in turn could negatively effect steroidogenesis in the testis. Although our data point toward a primary Leydig cell dysfunction, as evidenced by the dissociation between circulating LH and testosterone levels, further studies are required to (i) conclusively determine whether the observed steroidogenic impairment arises from local macrophage–Leydig cell interactions within the testicular microenvironment, or, at least in part, involves higher-order immune–endocrine mechanisms acting at the level of the HPG axis, and (ii) elucidate the mechanistic pathways through which macrophages regulate Leydig cell steroidogenic capacity.

Overall, depletion of CX3CR1^+^ macrophages significantly disrupted immune regulation under both steady-state and inflammatory conditions, particularly in the epididymis, where CX3CR1^hi^ macrophages are integral to mucosal immunity. While systemic effects resulting from testicular dysfunction (particularly regarding steroidogenesis) cannot be excluded, the focal, segment-specific nature of the epithelial damage observed in the epididymis suggests that local mechanisms, likely involving macrophage-epithelial interactions, play a significant role. More importantly, 30 days after Dtx-treatment testosterone levels remain decreased while epithelial integrity is reconstituted, as are macrophage numbers. Intriguingly, although germ cell development and maturation were disrupted in both the testis and epididymis, these effects were largely reversible. This suggests that while these processes are highly sensitive to homeostatic imbalance, tissue-intrinsic mechanisms are sufficient to restore local homeostasis, in part by recruiting circulating immune cells to repopulate the niche. Identifying the factors driving this immune restoration remains a key area for future research. Under inflammatory conditions frequently associated with persistent fertility issues in epididymitis patients, intraepithelial macrophages in the epididymis appear to play a central role in modulating the onset of inflammation. As such, they represent promising targets for therapeutic intervention aimed at improving clinical outcomes and preserving male fertility in immunological-based disorders.

## Supplementary Material

Supplementary Figure 3

Supplementary Figure 4

Supplementary Figure 8

Supplementary Figure 2

Supplementary Figure 5

Supplementary Figure 6

Supplementary Figure 1

Supplementary Figure 7

## Figures and Tables

**Table 1:** Scoring system of histopathological alterations after UPEC infection.

Score	Testis histopathology	Epididymis histopathology
0	no histological alterations, normal tissue architecture	
1	Scattered/focal mild histological alterations incl. mild reduction of tubular diameter, interstitial immune cell infiltration, overall normal spermatogenesis and intact seminiferous epithelium (SE)	Scattered/focal mild histological alterations) incl. mild reduction of luminal diameter and epithelial height), mild interstitial immune cell infiltration (scattered leukocytes up to 10% of interstitial space)
2	Confluent mild histological alterations, scattered apoptotic immature germ cells in SE, scattered interstitial immune cell infiltrations (approx. 10% of interstitial space)	Confluent mild histological alterations, mild interstitial immune cell alterations (10–30% of interstitial space)
3	Confluent moderate histological alterations, widespread disruption of SE incl. numerous apoptotic and exfoliated immature germ cells, interstitial immune cell infiltrations (approx. 20–30% of interstitial space)	Moderate histological alterations with focal epithelial lesions and moderate interstitial immune cell infiltration (30–50% of interstitial space filled with leukocytes)
4	Severe histological alterations, significantly smaller testis, moderate interstitial immune cell infiltrations (approx. 30–50% of interstitial space), strong disruption of spermatogenesis and confluently reduced tubular diameter	Moderate confluent epithelial damage incl. reduced duct diameter, cytoplasmic vacuolation, pyknotic nuclei, exfoliated epithelial cells in the lumen, reduced sperm volume in the duct, moderate immune cell infiltration (50–90% of interstitial space filled with leukocytes
5	Severe histological alterations, complete loss of normal testicular architecture, absence of meiotic germ cells, atrophy of SE, significantly reduced testis weight, extensive immune cell infiltration (up to 80%)	Severe epithelial damage, incl. confluent loss of epithelial integrity, extravasated sperm and sperm granuloma, presence of ‘ductal ghosts’/ duct stenosis, severe immune cell infiltrations (>90% of interstitial space filled with leukocytes) and fibrosis

## Data Availability

The transcriptomic datasets generated during this study were deposited in the NCBI Gene Expression Omnibus (GEO, accession no. GSE309348).

## Appendix A

**Table 2:** Key resource table.

Reagent type/species/Resource	Designation	Source or reference	Identifiers
Mouse (*Mus musculus*, male)	C57BL/6J wild type	Charles River	JAX ID: 000664
Mouse (*Mus musculus*, male)	*Cx3cr1*^CreER^ (B6.129P2(C)-*Cx3cr1*^*tm2.1(cre/ERT2)Jung*^/J	Yona et al. 2013(42)	JAX ID: 020940
Mouse (*Mus musculus*, male)	*Rosa26*^iDTR^ (C57BL/6-*Gt(ROSA)26Sor*^*tm1(HBEGF)Awai*^/J	Buch et al. 2005(43)	JAX ID: 007900
Mouse (*Mus musculus*, male)	*Rosa26*^tdTomato^ (B6.Cg-*Gt(ROSA)26Sor*^*tm9(CAG-tdTomato)Hze*^/J	Madisen et al. 2010(44)	JAX ID: 007909
Uropathogenic *Escherichia coli* (UPEC)	CFT073	Welch et al. 2002(45)	NCBI: txid19.9310
Antibody	BUV 563 anti-mouse/human (rat monoclonal	BD Bioscience	Cat. No. 741242 RRID: AB_3685254
Antibody	BUV 737 anti-mouse CD3e (rat monoclonal)	BD Bioscience	Cat. No. 564380 RRID: AB_2738781
Antibody	BUV 805 anti-mouse CD45 (rat	BD Bioscience	Cat. No. 568336 RRID: AB_3684191
Antibody	Brilliant Violet 421 anti-mouse CD163 (rat monoclonal)	BioLegend	Cat. No. 155309 RRID: AB_2814063
Antibody	Brilliant Violet 570 anti-mouse Ly-6G/Ly-6C (GR-1) (rat monoclonal)	BioLegend	Cat. No. 108431 RRID: AB_10896783
Antibody	Brilliant Violet 750 anti-mouse CD19 (rat monoclonal)	BioLegend	Cat. No. 115,561 RRID: AB_2813978
Antibody	Brilliant Violet 785 anti-mouse I-A/I-E (MHC II)(rat monoclonal)	BioLegend	Cat.No. 107645 RRID: AB_2565977
Antibody	FITC anti-mouse CCR2 (rat monoclonal)	BioLegend	Cat.No. 150608 RRID: AB_2616980
Antibody	PE/Cy7 anti-mouse CX3CR1 (mouse monoclonal)	BioLegend	Cat.No. 149016 RRID: AB_2565700
Antibody	APC/Cy7 anti-mouse F4/80 (rat monoclonal)	BioLegend	Cat.No. 123118 RRID: AB_893477
Antibody	Phospho-Tyrosine (4G10) Mouse monoclonal antibody	Cell Signaling	Cat.No. 96215 RRID: AB_3096295
Antibody	acetylated α-tubulin (Lys40) rabbit monoclonal antibody	Cell Signaling	Cat.No. 5335 RRID: AB_10544694
Antibody	Anti-F4/80 rat monoclonal antibody	Bio-Rad	Cat.No. MCA497G RRID: AB_872005
Antibody	Anti-V-ATPase B1 subunit	Battistone labPaunescu et al. 2010 (54)	in house
Antibody	Anti-Aquaporin 9 antibody	Battistone labPastor-Soler et al. 2001(55)	in house
Antibody	Anti-cleaved caspase-3 (ASP175)	Cell Signaling	Cat.No. 9664 RRID: AB_2070042
Antibody	Anti-CD206	Abcam	Cat.No. ab64693 RRID: AB_1523910
Antibody	Anti-MHC-II (M5/114.15.2)	BioLegend	Cat.No. 107601 RRID: AB_313316
Antibody	Alexa Fluor 594 anti-mouse CD326 (Ep-CAM) antibody	BioLegend	Cat.No. 118222 RRID: AB_2563322
Antibody	Goat anti-rabbit IgG (H + L) Cross-Adsorbed Secondary Antibody, Alexa Fluor 488	Invitrogen	Cat.No. A-11008 RRID: AB_143165
Antibody	Donkey anti-mouse IgG (H + L), Highly Cross-Absorbed Secondary Antibody, Alexa Fluor 647	Invitrogen	Cat.No. A-31571 RRID: AB_162542
Antibody	Goat anti-rat IgG (H + L), Cross-adsorbed secondary antibody, Alexa Fluor 546	Invitrogen	Cat. No. A-11081 RRID: AB_141738
Antibody	Goat anti-rat IgG (H + L), Alexa Fluor 647	Abcam	Cat. No. ab150159 RRID: AB_2566823
Antibody	Donkey anti-chicken IgY, Alexa Fluor 488	Jackson ImmunoResearch	Cat.No. 703-545-155 RRID: AB_2340375
Antibody	Donkey anti-rabbit IgG, Alexa Fluor 647	Jackson ImmunoResearch	Cat.No. 711-606-152 RRID: AB_2340625
Antibody	Donkey anti-rabbit IgG, Alexa Cy3	Jackson ImmunoResearch	Cat.No. 711-165-152 RRID: AB_2307443
Antibody	Anti-mouse IgG (H + L), HRP conjugate	Promega	Cat. No.W4021 RRID: AB_430834
Other (substrate)	1-Step Ultra TMB-ELISA Substrate Solution	Thermo Fisher Scientific	Cat. No. 34028
Other (dyes)	DAPI	Invitrogen	Cat.No. D1306
Other (dyes)	APC DNA dye DRAQ7	BD Bioscience	Cat.No. 564904
Other (dyes)	Peanut Agglutinin (PNA) FITC-conjugated	Sigma-Aldrich	Cat.No.L7381
Other	ProLong Antifade Gold w/o DAPI	Invitrogen	Cat.No. P36930
Chemical compound	2 N sulfuric acid (H_2_SO_4_)	Merck	Cat. No. 1603131003
Chemical compound	RIPA Buffer	Merck	Cat. No. R0278
Chemical compound	phosSTOP Phosphatase Inhibitor	Roche	Cat. No. 4906845001
Chemical compound	cOmplete EDTA-free Protease Inhibitor	Roche	Cat. No.11873580001
Chemical compound	Collagenase A	Roche	Cat.No. 10103578001
Chemical compound	DNase I	Sigma	Cat.No. D4513
Commercial kits and assays	RNeasy Mini Kit	Qiagen	Cat.No. 74004
Commercial kits and assays	RNase-free DNase Kit	Qiagen	Cat.No. 79254
Commercial kits and assays	Mouse Testosterone ELISA Kit	Crystal Chem	Cat.No. 80552
Commercial kits and assays	Mouse Testosterone ELISA Kit-Positive Control	Crystal Chem	Cat.No. 80553
Commercial kits and assays	Mouse Luteinizing Hormone ELISA Kit	Thermo Fisher Scientific	Cat No. EEL121
Other	TrueStain FcX (anti-mouse CD16/CD32) Antibody	BioLegend	Cat.No. 101320
Other	Leica Stellaris 5 Confocal Microscope	Leica	N/A
Other	Leica Thunder Imager 3D TissueMicroscope	Leica	N/A
Other	Leica DM750 microscope	Leica	N/A
Other	Leica Cryotome CM1850	Leica	N/A
Other	Leica Microtome RM2255	Leica	N/A
Other	BDSymphony S6 High-Parameter Cell Analyzer	BD	N/A
Software	FlowJo v10.10.0 with plugins DownSampleV3	BD Life Sciences	
Software	Adobe Illustrator	Adobe	
Software	GraphPad Prism 10	GrapgPad Prism Software	

## Appendix B. Supplementary data

Supplementary data to this article can be found online at https://doi.org/10.1016/j.mucimm.2026.01.011.
