# Supplementary figures and images for: Depletion of CX3CR1+ macrophages results in disrupted functionality and immune surveillance within epididymis and testis
Source: Mucosal Immunol. Author manuscript; Available in PMC 2026 Jul 4. (PMC13332447; doi:10.1016/j.mucimm.2026.01.011)

**A**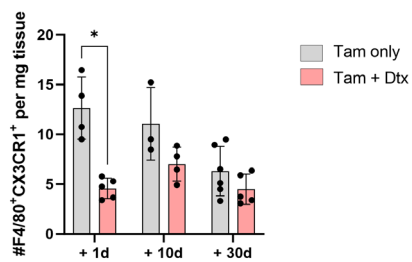**B**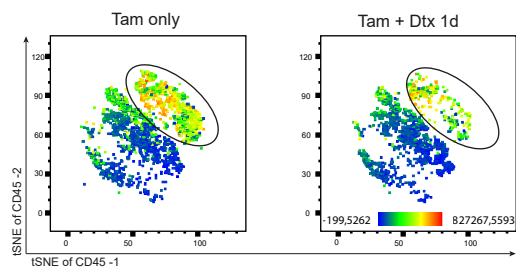**C**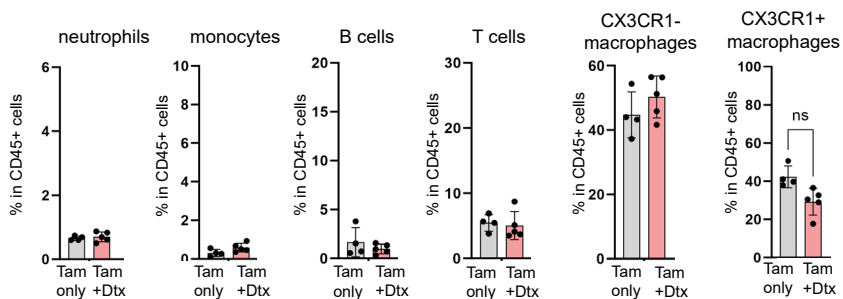**D**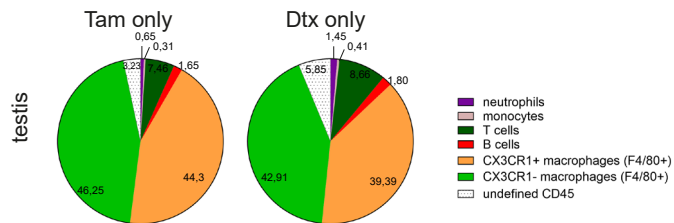

Supplement: Supplementary Figure 3 [file NIHMS2189516-supplement-Supplementary_Figure_3.pdf]

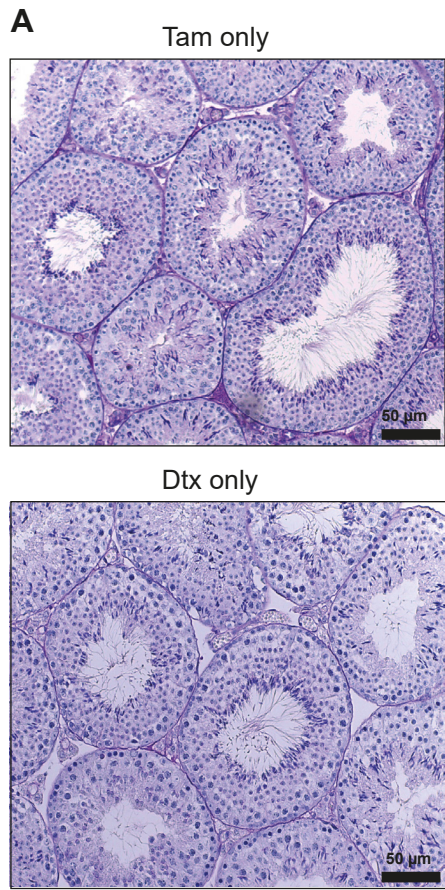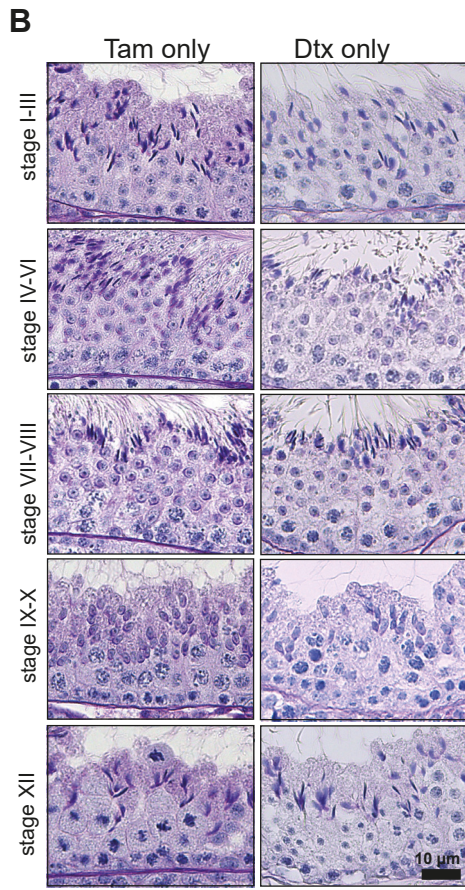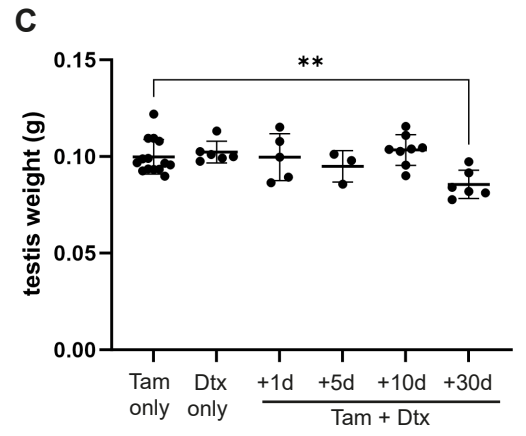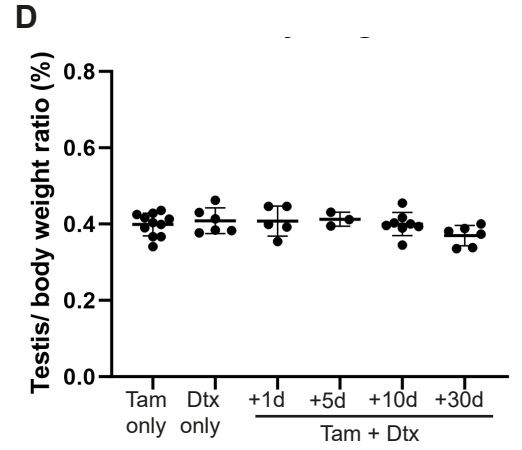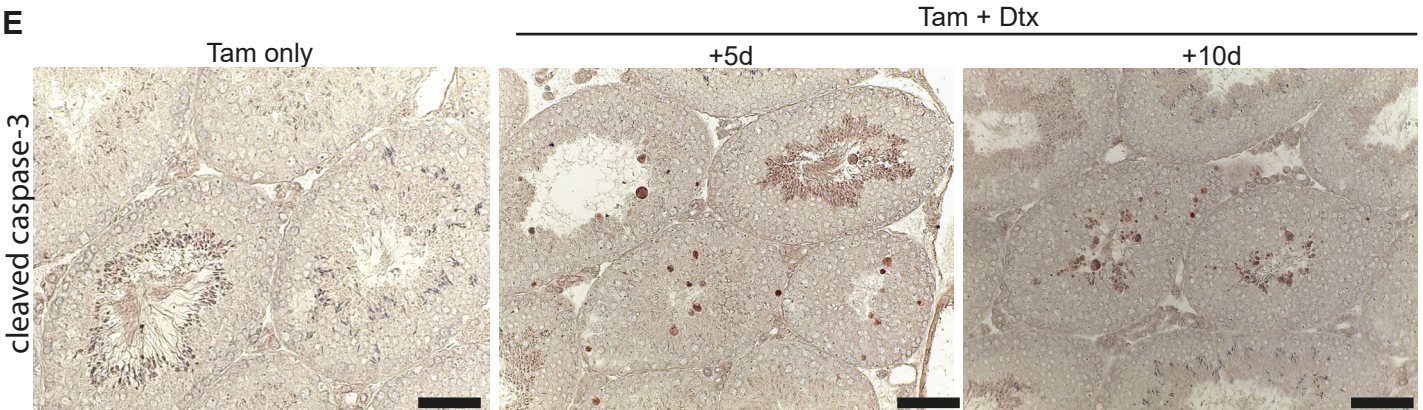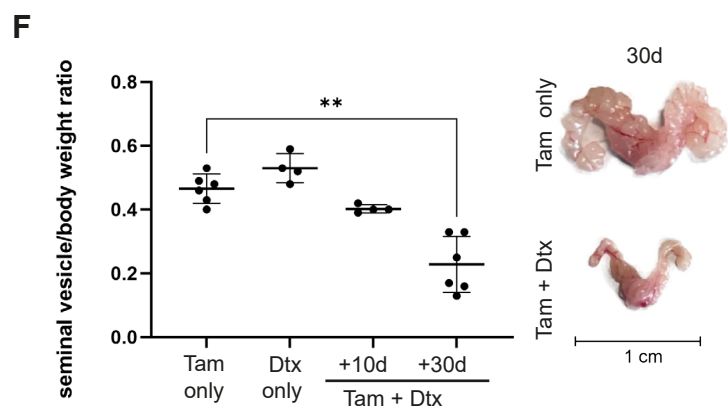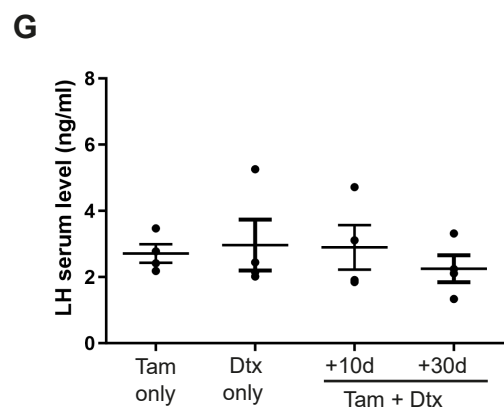

Supplement: Supplementary Figure 4 [file NIHMS2189516-supplement-Supplementary_Figure_4.pdf]

**A**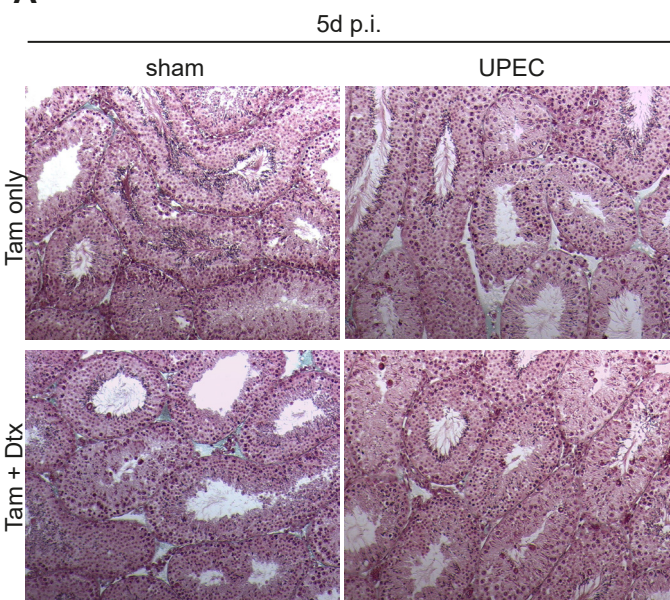**B**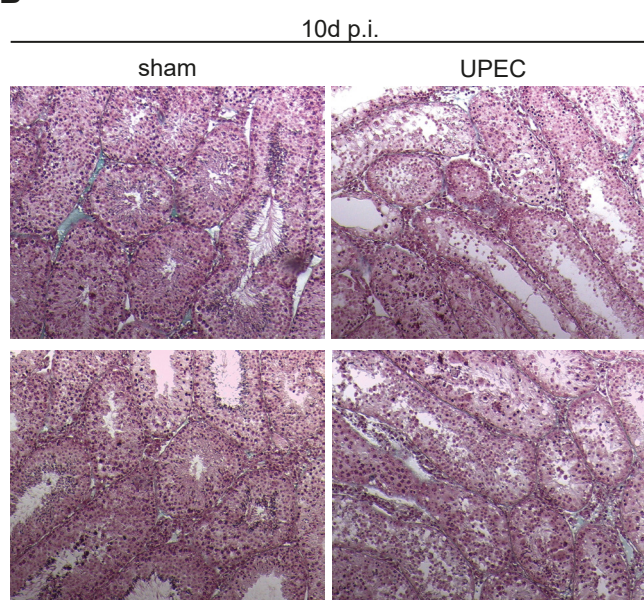**C**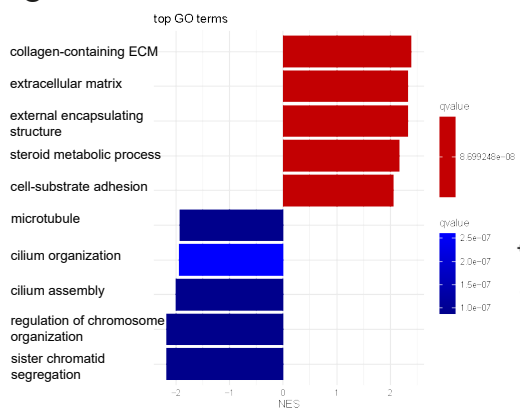**D**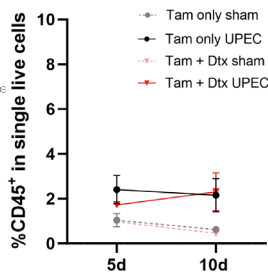**E**

Tam only UPEC (10d)

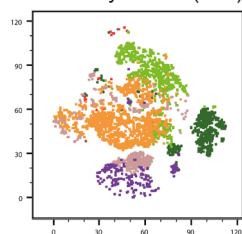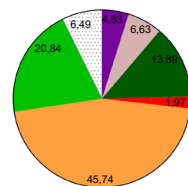

Tam + Dtx UPEC (10d)

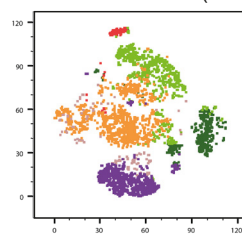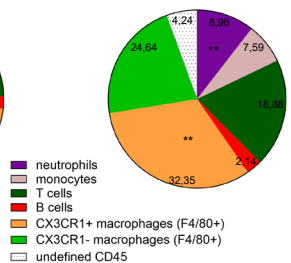

Supplement: Supplementary Figure 8 [file NIHMS2189516-supplement-Supplementary_Figure_8.pdf]

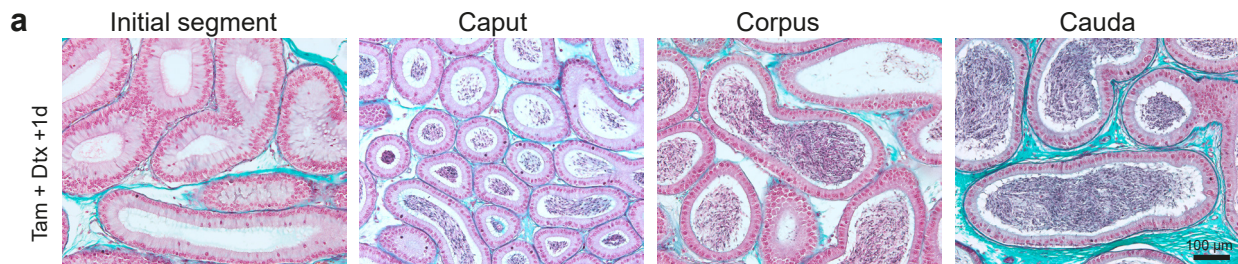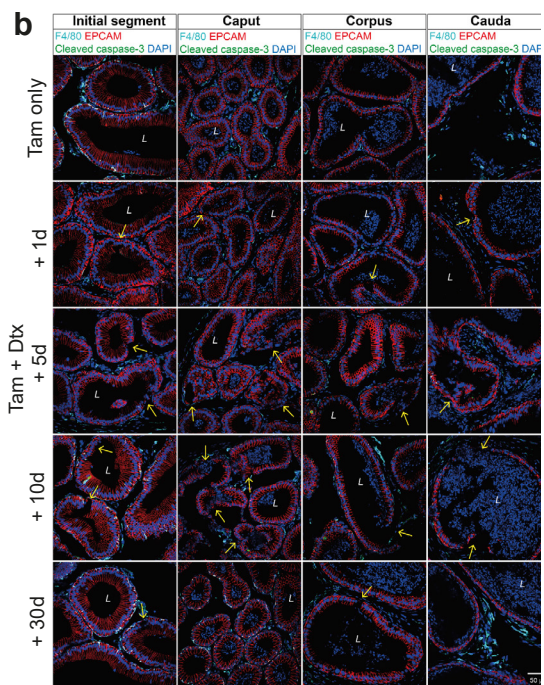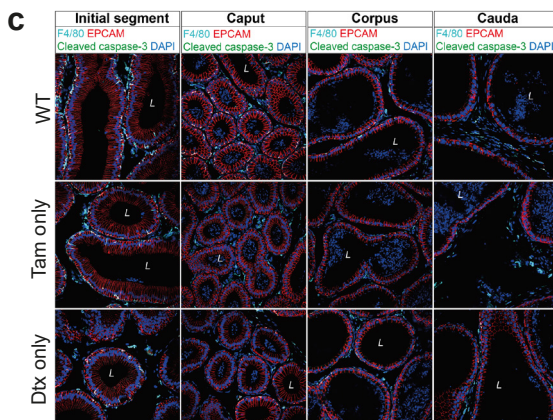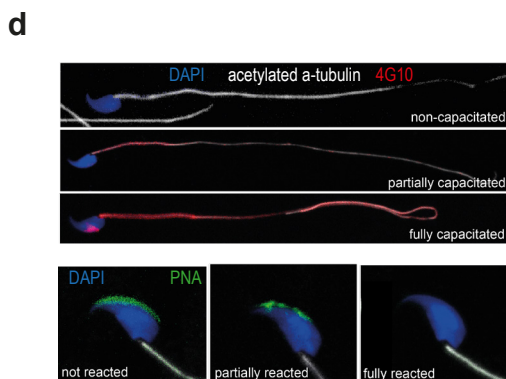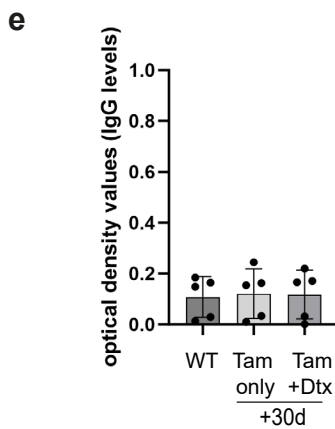

Supplement: Supplementary Figure 2 [file NIHMS2189516-supplement-Supplementary_Figure_2.pdf]

**A**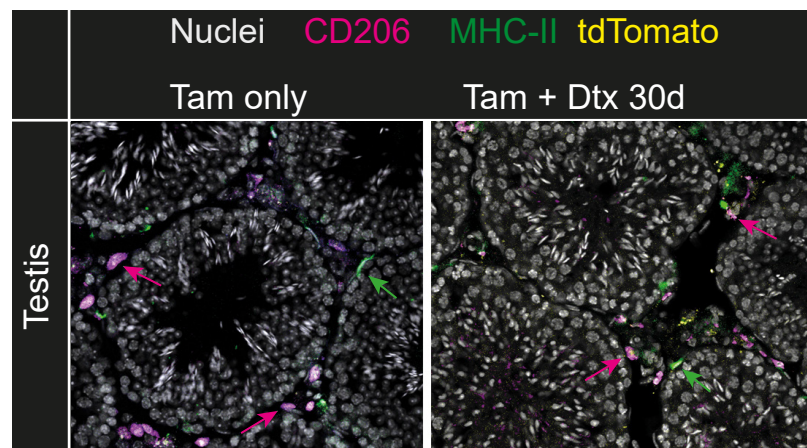**B**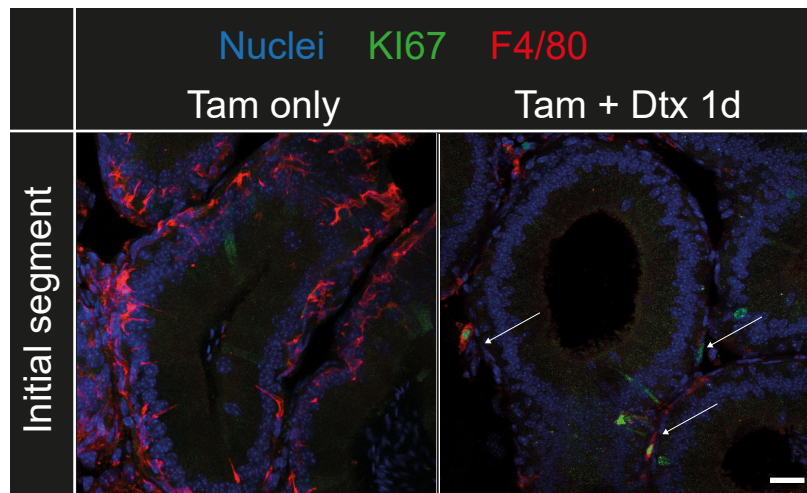

Initial segment

Caput

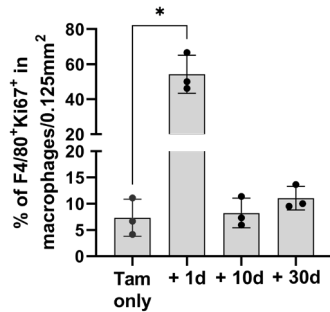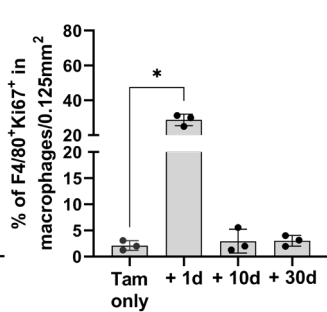

Corpus

Cauda

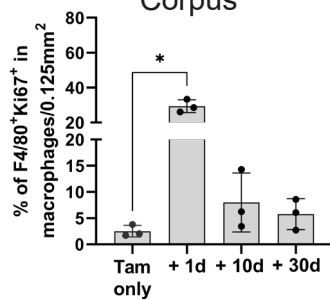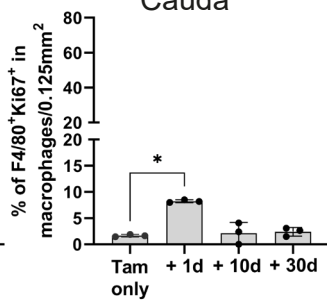**C**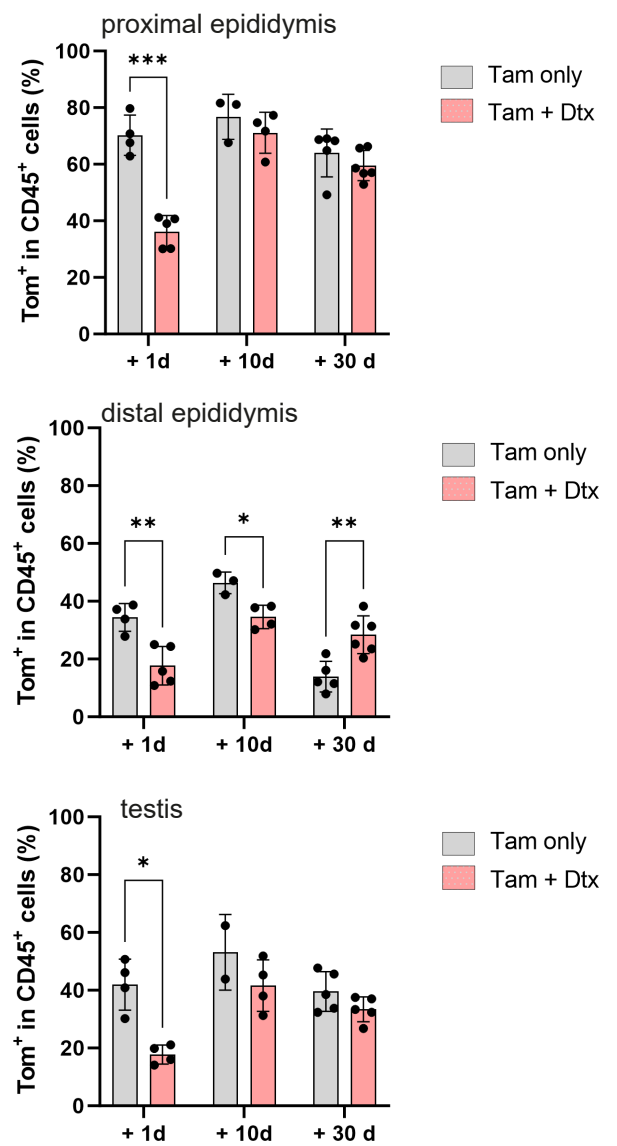

Supplement: Supplementary Figure 5 [file NIHMS2189516-supplement-Supplementary_Figure_5.pdf]

A

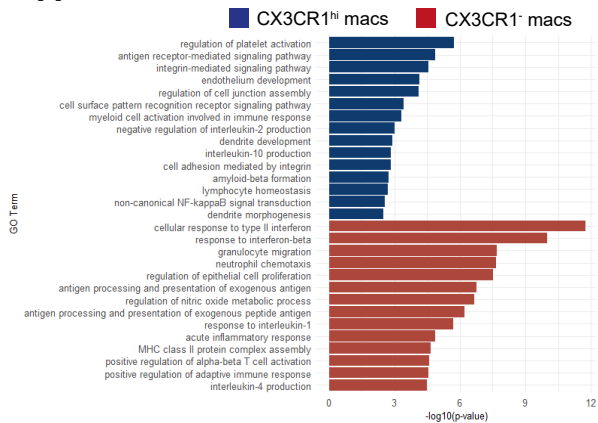

B

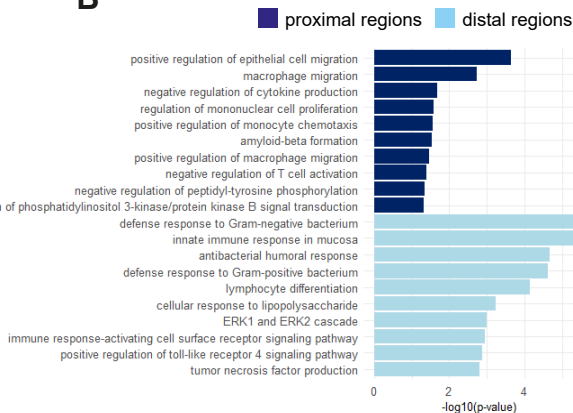

C

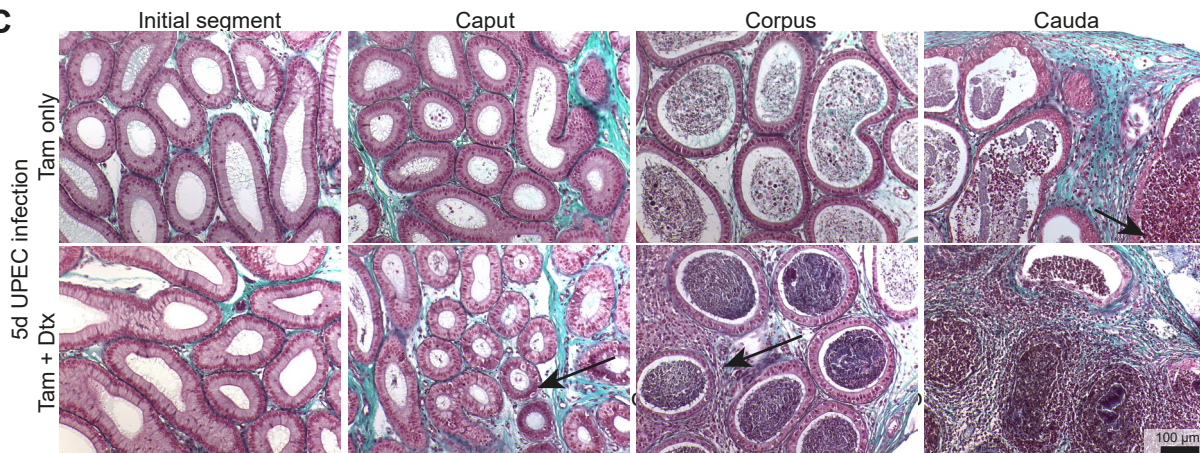

D

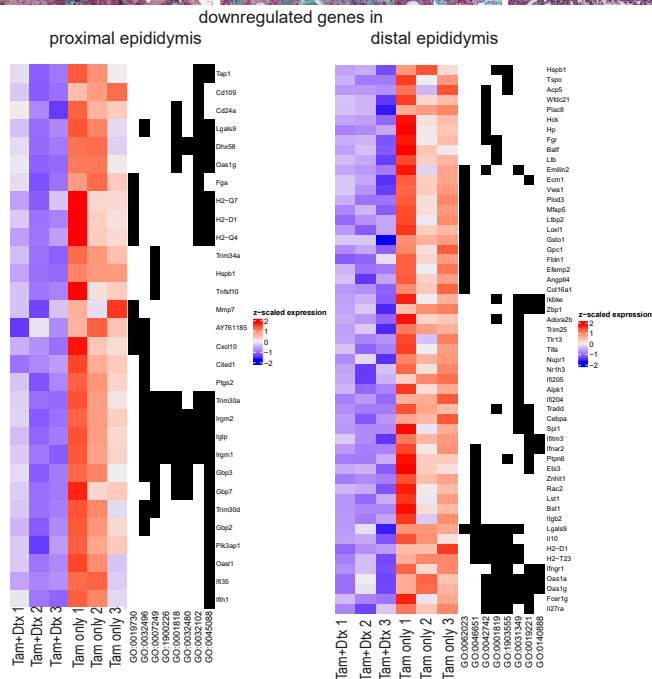

Supplement: Supplementary Figure 6 [file NIHMS2189516-supplement-Supplementary_Figure_6.pdf]

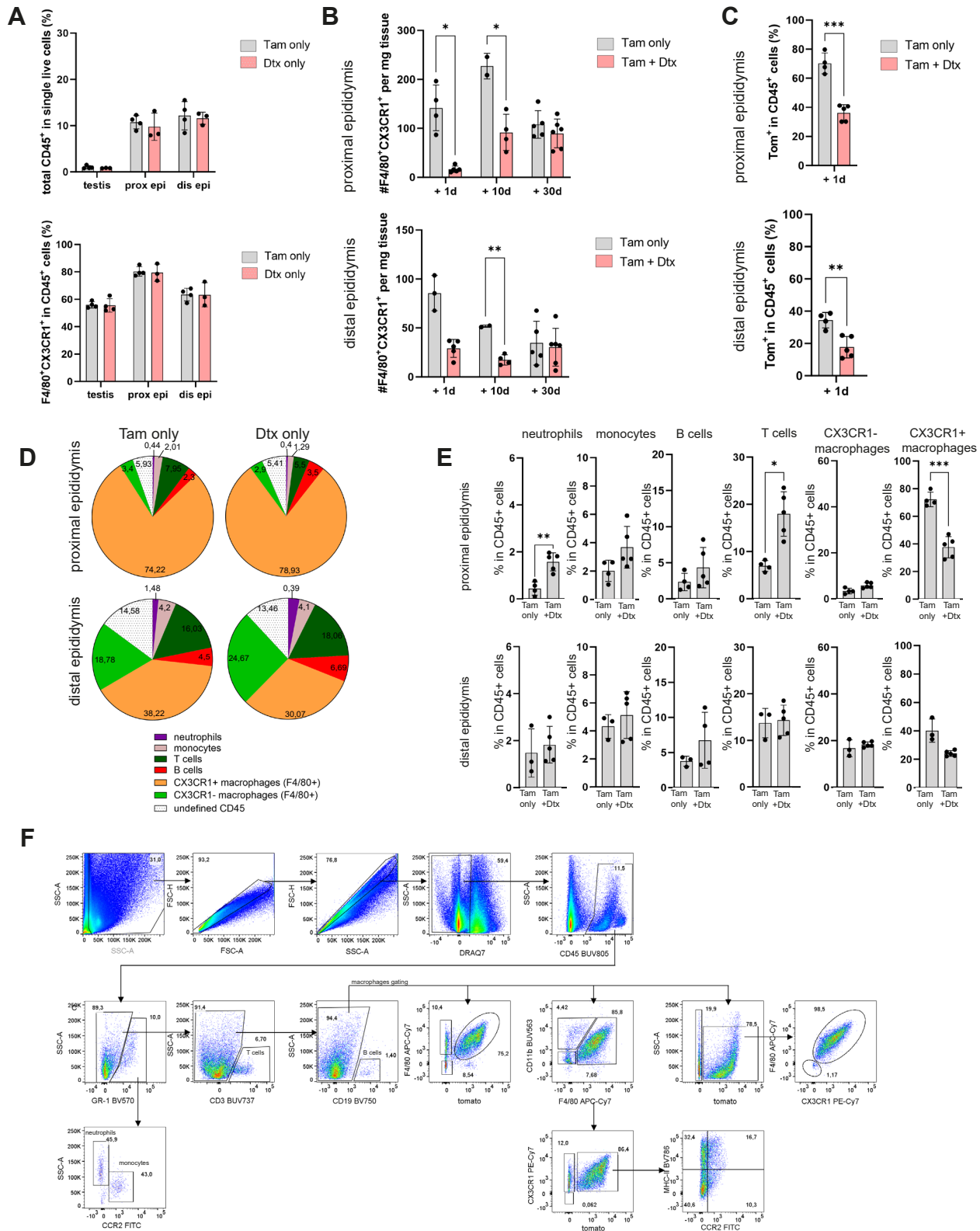

Supplement: Supplementary Figure 1 [file NIHMS2189516-supplement-Supplementary_Figure_1.pdf]

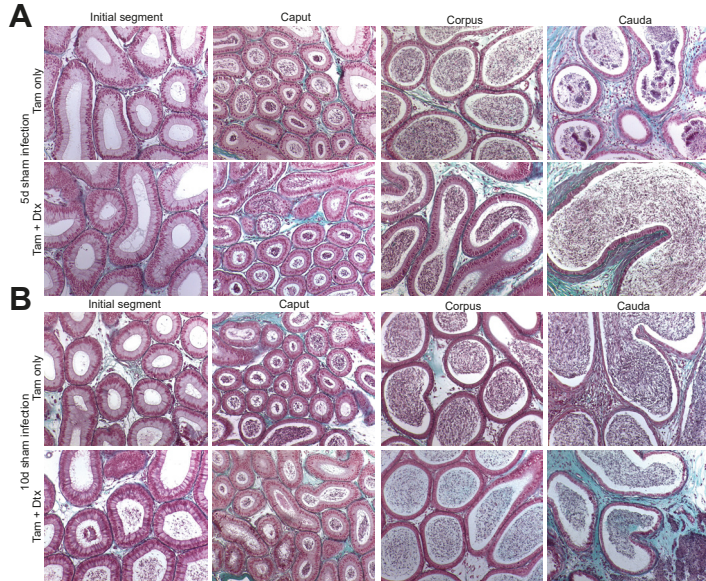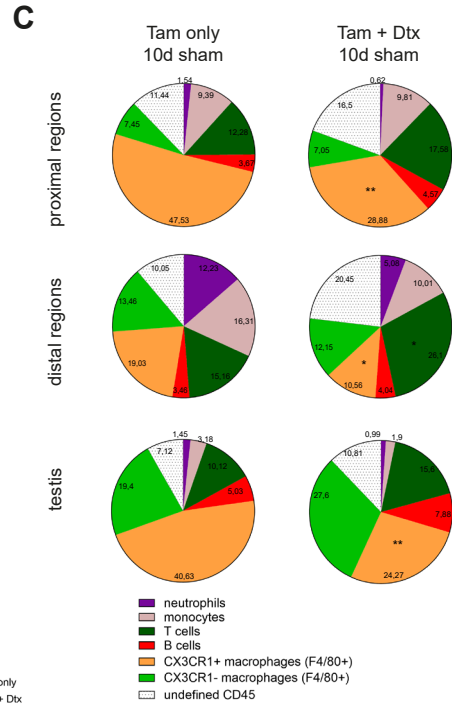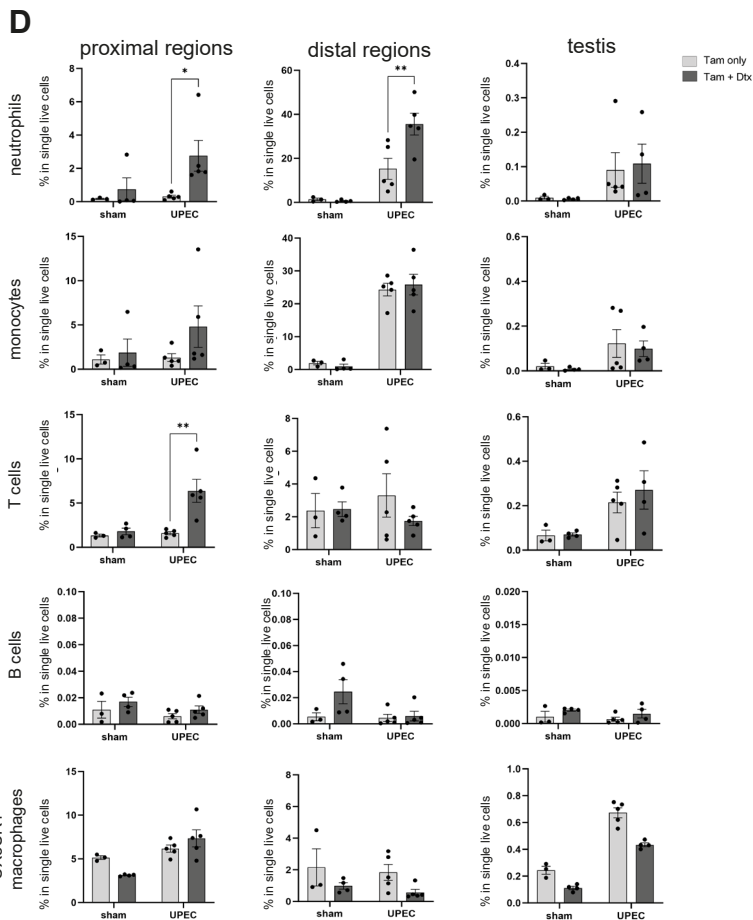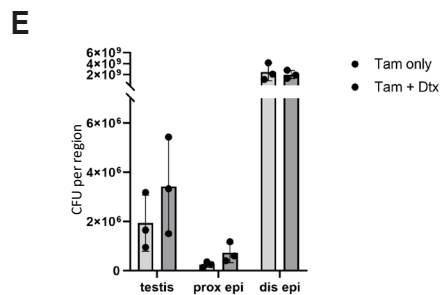

Supplement: Supplementary Figure 7 [file NIHMS2189516-supplement-Supplementary_Figure_7.pdf]
